# Investigation of Anti‐Apoptotic Effects and Mechanisms of Astragaloside IV in a Rat Model of Cerebral Ischemia–Reperfusion Injury

**DOI:** 10.1111/cns.70209

**Published:** 2025-01-07

**Authors:** Li Yu, Weifeng Jin, Defang Deng, Yiru Wang, Qianqian Chen, Yangyang Zhang, Haitong Wan, Yunxiang Chen, Ying Chen, Yu He, Lijiang Zhang

**Affiliations:** ^1^ Qingshan Lake Science and Technology Innovation Center Hangzhou Medical College Hangzhou China; ^2^ School of Basic Medical Sciences Zhejiang Chinese Medical University Hangzhou China; ^3^ Key Laboratory of Drug Safety Evaluation and Research of Zhejiang Province, Center of Safety Evaluation and Research Hangzhou Medical College Hangzhou China; ^4^ School of Pharmacy Zhejiang Chinese Medical University Hangzhou China; ^5^ Faculty of Chinese Medicine Macau University of Science and Technology Macao China

**Keywords:** Apoptosis, Astragaloside IV, Cerebral ischemia–reperfusion injury, JNK/Bid, SP600125

## Abstract

**Background:**

Ischemic stroke is a prevalent and life‐threatening cerebrovascular disease that is challenging to treat and associated with a poor prognosis. Astragaloside IV (AS‐IV), a primary bioactive component of *Astragali radix*, has demonstrated neuroprotective benefits in previous studies. This study aimed to explore the mechanisms through which AS‐IV may treat cerebral ischemia–reperfusion injury (CIRI).

**Methods:**

Network pharmacology was employed to identify key targets and pathways of AS‐IV in CIRI therapy, combined with molecular docking to predict binding affinity. Male Sprague–Dawley rats were randomly assigned to sham, MCAO/R, AS‐IV, SP600125 (JNK inhibitor), AS‐IV + SP600125, and 3‐n‐Butylphthalide (NBP) groups. Neurobehavioral deficits were assessed, and brain tissue damage was visualized through 2,3,5‐triphenyltetrazolium chloride, H&E, and TUNEL staining. Immunohistochemistry was employed to detect CytC‐ and caspase‐3‐positive cells, while Western blotting, qPCR, and ELISAs were used to analyze apoptosis‐related markers.

**Results:**

A total of 48 key targets of AS‐IV predicted to be involved in the treatment of CIRI were identified, enriched in 136 pathways. AS‐IV was effectively bound to the top five targets from 48 targets, and those associated with the c‐Jun N‐terminal kinase (JNK)/Bid pathway, with binding energy values below −5.0 kJ·mol^−1^. JNK inhibition reduced infarcted brain areas, improved neurological function, reduced pathological brain tissue damage, and inhibited apoptosis, with AS‐IV achieving similar neuroprotective effects. Both AS‐IV and SP600125 reduced p‐JNK, Bid, CytC, Apaf‐1, caspase‐3, and cleaved caspase‐3 levels in rats while decreasing CytC, caspase‐3, and caspase‐9 levels in serum.

**Conclusion:**

AS‐IV may suppress apoptosis partly through the modulation of JNK/Bid signaling, exerting neuroprotective effects. These findings support the potential development of AS‐IV‐based therapies for stroke treatment.

## Graphical abstract

AS‐IV is one of the primary bioactive components of *Astragali radix* with neuroprotective benefits. This study explored the possible mechanisms of AS‐IV treatment of CIRI through network pharmacology and experiment verification, which was linked to the JNK/Bid pathway.

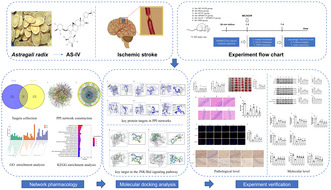

**Abbreviations**
AS‐IV: Astragaloside IV
BBB: blood–brain barrier
BP: biological process
CC: cellular component
CCA: common carotid artery
CIRI: cerebral ischemia–reperfusion injury
ECA: external carotid artery
GO: gene ontology
H&E: hematoxylin and eosin
ICA: internal carotid artery
IS: ischemic stroke
JNK: c‐Jun N‐terminal kinase
KEGG: Kyoto encyclopedia of genes and genomes
MCAO/R: middle cerebral artery occlusion/reperfusion
MF: molecular function
NBP: 3‐n‐Butylphthalide
PPI: protein–protein interaction
SD: Sprague–Dawley
TCM: traditional Chinese medicine
TTC: 2,3,5‐triphenyltetrazolium chloride

## 1 Introduction

Stroke is the second leading cause of global mortality and the third most common cause of disability [1], resulting in an estimated 5.5 million deaths per year [2]. Stroke is also the most common cause of death and disability in China [3]. Ischemic stroke (IS) cases account for 80% of all cases [4]. Advances in therapeutic technologies have reduced stroke‐related morbidity and mortality, but the disease continues to have devastating effects on families and societies throughout the world [5]. Cerebral ischemia–reperfusion injury (CIRI) is a condition that arises when the restoration of blood flow and oxygen availability following ischemia results in further damage to brain function following current interventional treatments recommended to treat IS (including mechanical thrombectomy and drug thrombolysis) [6, 7, 8]. CIRI involves multiple forms of cell death, including apoptosis, necroptosis, ferroptosis, pyroptosis, and autophagy, all contributing to brain tissue damage [9].

Apoptosis is a primary mode of cell death in IS, arising through an endogenous pathway driven by mitochondria‐mediated caspase activation, an exogenous pathway driven by the activation of intracellular caspases in response to signaling through surface death receptors including Fas, and/or an ER stress pathway [10, 11, 12]. Apoptotic activity can be activated in both physiological and pathological settings, including cellular development, replacement, and stress. The incidence of CIRI exposes the brain tissue to ischemia and hypoxia, which directly activate apoptosis‐related genes. The combined effects of several factors can also contribute to apoptotic induction, including ER stress, EAA/ROS mechanisms, Ca^2+^ overload, inflammation, and abnormal energy metabolism resulting from mitochondrial damage. As a key MAPK family member, c‐Jun N‐terminal kinase (JNK) is a key mediator of intracellular signaling involved in neuroinflammation, neuronal apoptosis, memory formation, and brain repair [13], all involving JNK activation. The JNK pathway is among the most important apoptosis‐related pathway. Under stress conditions, the translocation of JNK from the cytosol to the nucleus can activate the JNK cascade, ultimately acting on members of the Bcl‐2 family to control apoptosis. Liu et al. [14] determined that JNK/caspase‐3 pathway inhibition was sufficient to protect against CIRI in mice.

Current IS management focuses on vascular recanalization to restore blood flow and preserve neuronal integrity. However, the timeframe during which recanalization is effective is limited, and it can result in intracranial hemorrhage and neurovirulence. Neuroprotective strategies are thus the primary focus of novel drug development efforts [15]. Traditional Chinese medicine (TCM) practices have been repeatedly demonstrated in preclinical and clinical studies to exert beneficial effects to prevent and treat IS. *Astragali radix* is cultivated primarily in China and neighboring countries, with major cultivation areas including Shanxi, Heilongjiang, and Inner Mongolia. As an important medicinal and edible herb, it has multiple effects, such as tonifying qi and stabilizing the surface, strengthening the heart, and lowering blood pressure. In recent years, the planting area of *Astragali radix* has been expanding year by year, and its industry has become an important pillar of rural revitalization. Meanwhile, with the widespread promotion of medicinal and edible Chinese herbal medicines, *Astragali radix* has entered the era of deep processing. It is used in pharmaceuticals, health products, and food production.

**FIGURE 1:**
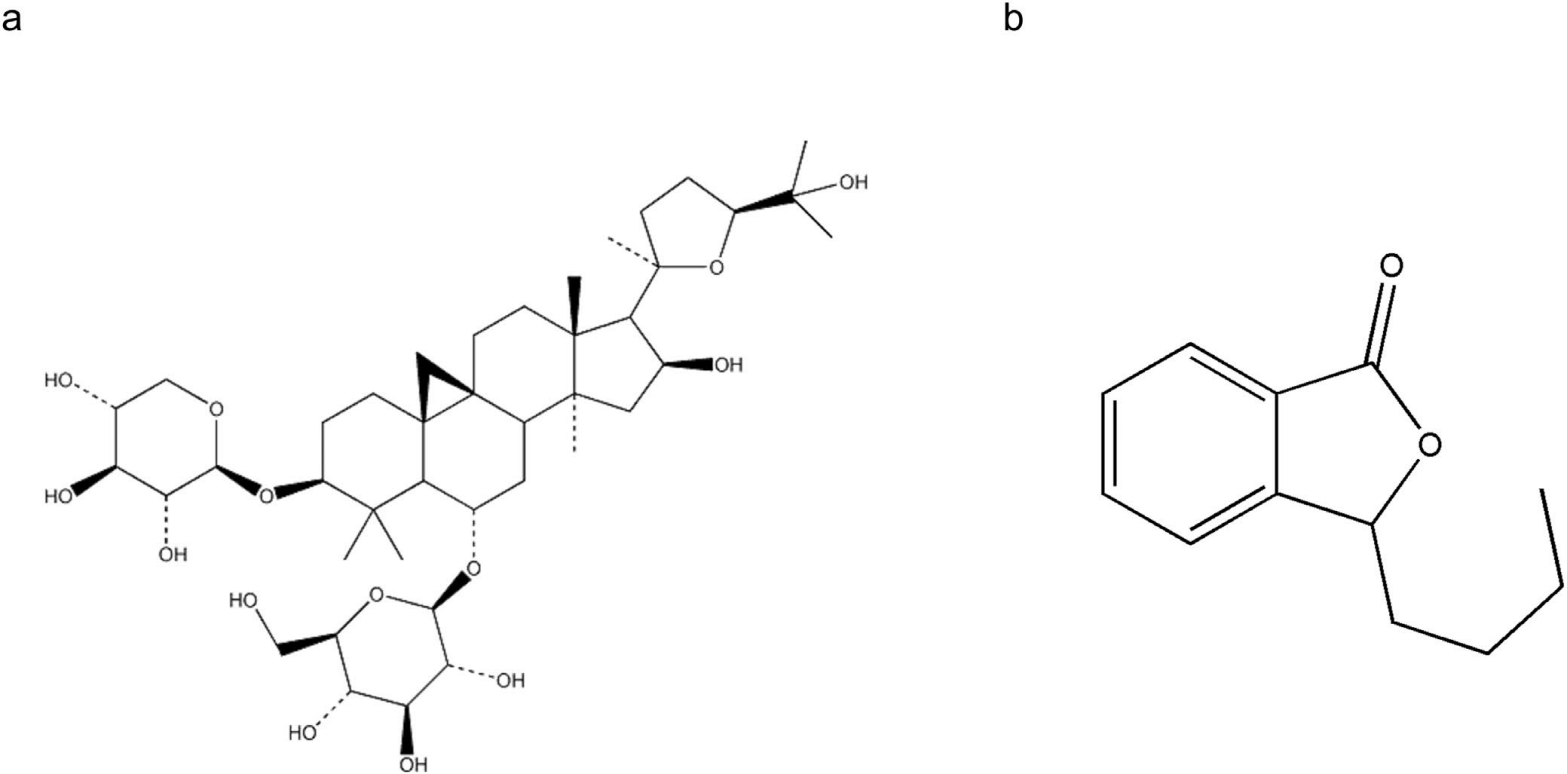
The chemical structure of Astragaloside IV (a) and 3‐n‐Butylphthalide (b).

Astragaloside IV (AS‐IV) is the primary bioactive ingredient derived from *Astragali radix* and the key ingredient associated with *Astragali radix* quality control in the Chinese Pharmacopeia. The formula for AS‐IV is C_14_H_68_O_14_ (Figure 1a). It is commonly used in China to prevent or treat cardiovascular and cerebrovascular diseases [16]. AS‐IV has been shown to reduce the infarcted volume significantly and to facilitate improved post‐CIRI neurological recovery [17]. It also reportedly exhibits anti‐inflammatory, antioxidant, and anti‐apoptotic activities together with the ability to improve the blood–brain barrier (BBB), microcirculatory perfusion, and energy metabolism [16, 18, 19, 20, 21]. Research on AS‐IV's protective effects against CIRI is currently limited to cell lines and animal models, and the mechanisms through which it protects against adverse neurological outcomes remain uncertain.

Network pharmacology strategies rely on a multi‐drug, multi‐target, multi‐pathway framework to explore the mechanisms of action for different drugs based on available TCM, protein, gene, and other databases, establishing drug‐target‐disease networks [22]. Here, network pharmacology and molecular docking approaches were used to explore potential targets and molecular mechanisms through which AS‐IV can treat IS, providing a foundation for further research on how AS‐IV protects against CIRI. To elucidate the CIRI improvement situation following AS‐IV treatment and the association between the JNK pathway and apoptotic inhibition, 3‐n‐Butylphthalide (NBP, C_12_H_14_O_2_, Figure 1b) and the JNK inhibitor SP600125 were also employed in a rat model of CIRI.

## 2 Materials and Methods

### 2.1 Materials and Reagents

AS‐IV (≥ 98% purity, Cat.#DH0015‐0020, Lot.#DSTDH001502) from Desite, Chengdu, China, and SP600125 (Cat.#HY‐12041, Lot.#117,629) from MCE, USA, were used in this study. 2,3,5‐Triphenyltetrazolium chloride (TTC, Cat.#YRT8877, Lot.#BCCC4696) was from Sigma, Germany. Primers for the genes encoding JNK, CytC, Apaf‐1, caspase‐9, and caspase‐3 were from Sangon Biotech Biological Engineering Co. Ltd., Shanghai, China. JNK (Cat.#AF6319, Lot.#84b6974), p‐JNK (Cat.#AF3318, Lot.#36c5455), Bid (Cat.#DF6016, Lot.#82 m4880), CytC (Cat.#AF0146, Lot.#73c2522), Apaf‐1 (Cat.#AF0117, Lot.#29×2038), caspase‐3 (Cat.#AF6311, Lot.#76i4559), and cleaved caspase‐3 (Cat.#AF7022, Lot.#15z0096) antibodies were from Affinity, USA. ELISA kits specific for CytC (Cat.#MB‐1934A, Lot.#202,209), caspase‐3 (Cat.#MB‐7014A, Lot.#202,209), and caspase‐9 (Cat.#MB‐7254A, Lot.#202,209) were from Jiangsu Meibiao Biotechnology Co. Ltd., Jiangsu, China.

### 2.2 Network Pharmacology Analyses

#### 2.2.1 AS‐IV Targets Identification

The SMILES number and SDF file for AS‐IV were retrieved from the PubChem database (https://pubchem.ncbi.nlm.nih.gov/). This SMILES number was entered into the Swiss Target Prediction database (http://swisstargetprediction.ch/) to access a list of potential targets, deleting any targets with a probability of 0. The SDF file for AS‐IV was introduced into the PharmMapper database (https://www.lilab‐ecust.cn/pharmmapper/) for further target prediction efforts. Identified target proteins from these databases were pooled and uploaded to the UniProt database (https://www.uniprot.org/) to convert gene names for target proteins, yielding a final list of AS‐IV targets following deduplication.

#### 2.2.2 CIRI‐Related Targets Identification

CIRI‐related targets were identified using the Gene‐Cards database (https://www.genecards.org/) and OMIM database (https://www.omim.org/) with “cerebral ischemia–reperfusion injury” as the search term. After combining the targets from these two databases, deduplication was performed to yield a final list of disease targets.

#### 2.2.3 Protein–Protein Interaction (PPI) Network Generation and Key Targets Selection

The Venny2.1.0 platform (https://bioinfogp.cnb.csic.es/tools/venny/) was used to generate a Venn diagram for the AS‐IV and CIRI target genes, identifying the intersecting targets as potential AS‐IV targets relevant to CIRI treatment. STRING 11.0 database (https://version‐11‐0.string‐db.org/) was used to construct a PPI network for these targets, limiting the species to *Homo sapiens*, restricting the minimum required interaction score to 0.4, and hiding the broken protein nodes in the network. The resultant network was imported into Cytoscape 3.6 for topology analyses, sorting the results based on degree values. Targets with a degree value above the average were identified as key targets through which AS‐IV may act on CIRI.

#### 2.2.4 Functional Enrichment Analyses

Key AS‐IV targets involved in CIRI were imported into the DAVID database (https://david.ncifcrf.gov/), conducting gene ontology (GO) and Kyoto encyclopedia of genes and genomes (KEGG) functional enrichment analyses, restricting the species to *Homo sapiens*. GO terms included biological process (BP), molecular function (MF), and cellular component (CC) associated with key targets, with enriched GO terms and KEGG pathways being identified based on a significance threshold of *p* < 0.05. The top 20 KEGG pathways were visualized with a Bioinformatics platform (https://www.bioinformatics.com.cn/), as were the top 10 BP, MF, and CC terms.

#### 2.2.5 Molecular Docking

The SDF file for AS‐IV was imported into the Chem3D software, and energy optimization was performed. A file for AS‐IV in the pdb format was transformed with Open Babel 3.1.1 for subsequent docking. The RCSB PDB database (https://www.rcsb.org/) was accessed to obtain a file for the target protein in the pdb format. After using PyMol to remove ligands, metal ions, and water molecules from this structure, it was introduced into Autodock4.2 for hydrogenation, charge calculation, atomic type setting, and additional adjustments, followed by export in the pdbqt format. After pretreatment, the docking parameters were established, using AS‐IV as the ligand and target proteins as receptors. Molecular docking was performed with the Genetic Algorithm Parameters algorithm, and results were visualized with PyMol.

### 2.3 Experimental Animals

Specific pathogen‐free male Sprague–Dawley (SD) rats (260 ~ 300 g) were obtained from the Animal Laboratory Center of Zhejiang Chinese Medical University (Certificate Number: IACUC‐20220606‐05; Production license number: SCXK (Shanghai) 2018–0006). They were raised with free food/water access under a 12‐h light/dark cycle with standard conditions. After adaptive feeding for 7 days, rats were fasted for 12 h before surgery with free water access. The National Health Agency guidelines for the care and use of laboratory animals were adhered to when performing all animal studies.

### 2.4 Focal Middle Cerebral Artery Occlusion/Reperfusion (MCAO/R) Modeling

The MCAO/R model was established following the method described by Longa et al. [23]. After a 12 h fast, a suture/occlusion approach was used to perform the MCAO/R surgery. Briefly, rats were injected with atropine (0.04 mg/kg), followed 5 min later by an intraperitoneal Zoletil 50 (40 mg/kg) injection. Once anesthetized, these rats were fixed in the supine position, their neck hair was removed, the underlying skin was sterilized using alcohol, and an incision was made in the center of the neck. The right common carotid artery (CCA), external carotid artery (ECA), and internal carotid artery (ICA) were exposed, and 4–0 sutures were used to ligate the proximal CCA and ECA while using a mini arterial clamp to close the ICA. A V‐shaped notch was cut in the distal end of the CCA and the fork using ophthalmic scissors, and a bolt with a smooth ball at the head (50 mm long, 0.2 mm diameter) was advanced into the ICA. The arterial clamp was removed, and the bolt was advanced until the mark reached the fork, thereby blocking the MCA. Insertion was performed to 18–20 mm from the fork, ligating the ICA to fix the bolt. After ischemia for 1 h, the blot was removed, the notch was tightened, and the skin was sutured, allowing reperfusion to occur.

### 2.5 Animal Grouping and Treatment

**FIGURE 2:**
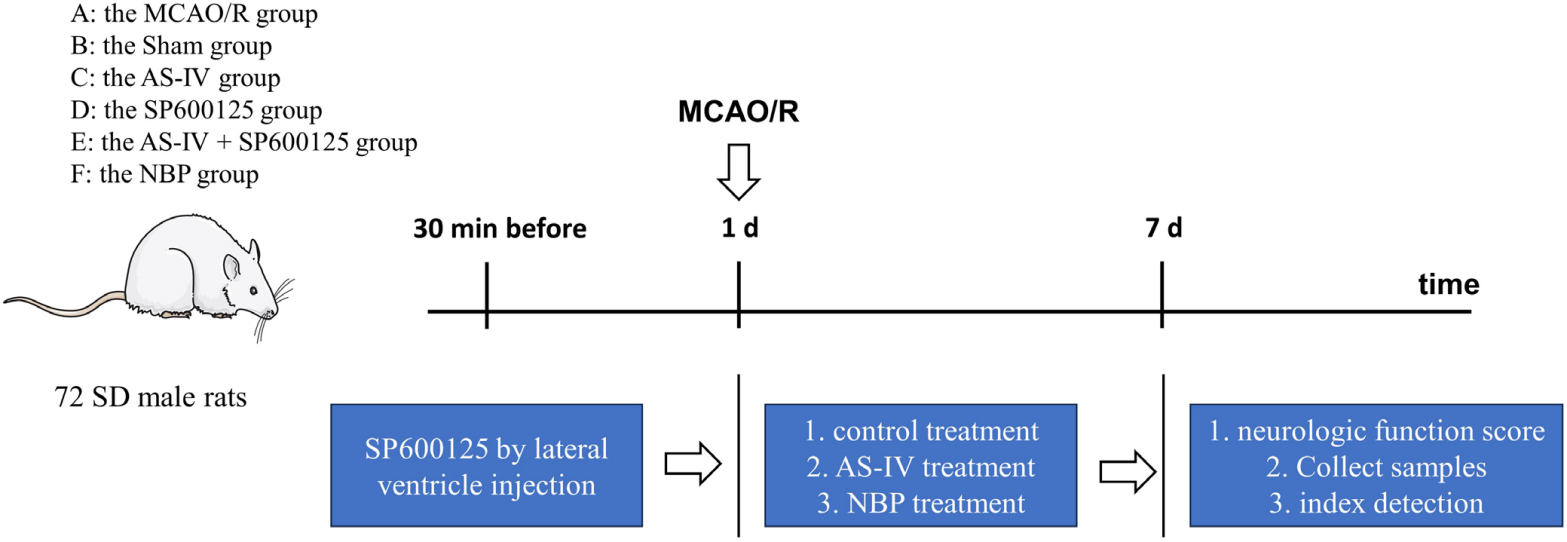
Experimental flowchart.

In total, 72 SD rats were randomized into the following groups: sham, MCAO/R, AS‐IV, SP600125, AS‐IV + SP600125, and NBP group. Rats in the NBP group received an intraperitoneal injection of NBP sodium chloride (10 mL/kg/d) [24]. Rats in the SP600125 groups received an injection of 10 μL SP600125 (10 mM in 0.1%DMSO) in the lateral ventricle 30 min before MCAO/R modeling. Rats in the AS‐IV groups received intragastric AS‐IV (40 mg/kg/d) [25, 26, 27]. Rats in the combination group received both AS‐IV and SP600125, as above. Sham and MCAO/R animals received an equivalent intragastric volume of normal saline. Treatments were administered daily for 7 days following the described protocols (Figure 2).

For SP600125 injection, the hair was removed from the heads of rats, which were deeply anesthetized and fixed to a stereotaxic apparatus. After cutting away the scalp, the fontanelle of the skull was located for injection. A lateral ventricular injection was then made to the lower right of this center point (1.5 mm right, 1.0 mm posteriorly). A microsyringe was inserted approximately 3.5 mm into the target site, injecting SP600125 at 1 μL/min with needle indwelling for 1–2 min post‐injection. The needle was then gradually removed every 2–3 min until fully removed, rats were removed from the stereotaxic apparatus, and the scalp was sutured.

### 2.6 Neurological Functional Scoring

Neurological function was assessed on day 7 post‐modeling using the Zea Longa and Garcia JH neurobehavioral scoring methods [28]. Zea Longa neurobehavioral scoring method: 0 points, no deficit; 1 point: inability to extend the opposite front paw when the tail is lifted; 2 points: turning to the opposite side during ambulation; 3 points: falling over on the opposite side during ambulation; and 4 points: no spontaneous walking, reduced consciousness. Modeling was deemed successful if rats showed forelimb ischemia (scores 1 ~ 3) upon awakening and showed no evidence of bleeding in the MCA. Garcia JH scoring method evaluates the degree of neurological impairment in rats from autonomous movement, body symmetry, forelimb stretching function, climbing movement, bilateral tactile sensation of the body, and bilateral whisker touch response (Score: 0 ~ 18). Higher scores indicated milder neurological impairment.

### 2.7 TTC Staining

Three rats per group were selected at random, and the whole brain was collected after decapitation, frozen at −20°C for 15 min, and cut along the coronal plane at 2 mm intervals before immersion in 2% staining solution for 15 min at 37°C in dark. Images were captured with a camera and analyzed using Image J software. The percentage of cerebral infarction volume was calculated as follows:

$$\begin{array}{c} \text{Percentage of cerebral infarction volume}\\ =\text{infarcted volume}/\text{total volume}\times 100\%. \end{array}$$

### 2.8 Histological Staining

Histopathological brain damage was assessed through hematoxylin and eosin (H&E) staining. At 7 days following MCAO/R modeling, rats were deeply anesthetized, the heart was sequentially perfused with saline and 4% paraformaldehyde, and the removed brain tissue was fixed in 4% paraformaldehyde. Following dehydration, paraffin embedding, and sectioning, brains were subjected to H&E staining. Following treatment with absolute ethanol and xylene until transparent, sections were sealed with neutral gum and imaged under a microscope, scoring them as follows: 0 points, normal tissue without damage; 1 point, slight tissue damage with increased tissue space; 2 points, slight tissue damage with increased tissue space and the mild neuronal necrosis and atrophy; 3 points, moderate damage with increases in tissue space and moderate neuronal necrosis and atrophy; and 4 points, severe tissue damage, including increases in tissue space together with extensive neuronal necrosis and atrophy.

### 2.9 TUNEL Staining

To assess apoptotic death in brain tissues, sections were dewaxed, rehydrated, and treated at room temperature with proteinase K, followed by membrane disruption and equilibration to room temperature, with TUNEL staining being performed at 37°C for 2 h. Sections were rinsed thrice with PBS (pH 7.4) and counterstained with DAPI. After three more washes, sections were sealed with an antifade mounting medium and imaged under a fluorescence microscope, with positive apoptotic nuclei appearing red. The apoptosis rate was calculated as follows:

$$\begin{array}{c} \text{Apoptosis rate}=\text{TUNEL}-\text{positive apoptotic cells number}/\\ \;\text{total cell number}\times 100\%. \end{array}$$

### 2.10 Immunocytochemistry

Paraffin‐embedded sections were treated with xylene for deparaffinization and hydrated with an ethanol gradient, after which they were treated using H_2_O_2_ to block endogenous peroxidase activity. Thermal antigen repair was performed in 1 mmol Tris‐EDTA buffer (pH 9.0), followed by three washes with PBS and blocking with serum. Antibodies specific for CytC or caspase‐3 were added overnight at 4°C. Following three more PBS washes, secondary antibodies were added for 30 min at 37°C for 30 min, followed by another round of washes and the addition of DAB for color development. After washing away the residual DAB, hematoxylin counterstaining was performed for 30 s. Following dehydration with an ethanol gradient, sections were treated with xylene, sealed using neutral gum, and imaged under a microscope. Brown or yellow particles indicated positive expression, whereas nuclei appeared blue.

### 2.11 Western Blotting

RIPA buffer was added to collected brain tissues, which were homogenized with a tissue homogenizer, lysed on ice for 30 min, and centrifuged (15 min, 12,000 rpm, 4°C) to extract proteins, which were then quantified using BCA. After separation by SDS‐PAGE, proteins were transferred to PVDF membranes. Blots were blocked for 2 h with 5% non‐fat milk at room temperature and then incubated with primary antibodies at 4°C overnight. After washing, blots were incubated with secondary antibodies for 90 min at room temperature with shaking, followed by immersion in ECL solution for 3 min. Blots were then imaged, with Image J being used for gray value analyses.

### 2.12 qPCR

A Trizol kit was used to harvest the total RNA from 50 to 100 mg of brain tissue, after which RNA purity and concentration were measured. cDNA was then prepared (42°C for 15 min, 85°C for 5 min), and qPCR was performed as follows: 95°C for 10 min, 95°C for 15 s, and 60°C for 60 s, 40 cycles. Primers used in this study are shown in Table 1.

**TABLE 1:** Primer sequences.

Gene	Forward primer	Reverse primer
JNK	AGGTAATGGATTTGGAGGAACG	TGACAGACGGCGAAGACGA
CytC	CTGTGGAAAAAGGAGGCAAGC	TCCATCAGGGTATCCTCTCCC
Apaf‐1	CGGCCCTGCGCATCTGATTCAT	GGGCGAACGACTAAGCGGGACAG
Caspase‐3	GACTGCGGTATTGAGACAGA	CGAGTGAGGATGTGCATGAA
Caspase‐9	CTGAGCCAGATGCTGTCCCATA	GACACCATCCAAGGTCTCGATGTA
GAPDH	GCTGAGAATGGGAAGCTGGT	GGTGGTGAAGACGCCAGTAG

### 2.13 ELISAs

After anesthetizing rats in each group, blood was collected from the heart, centrifuged (15 min, 4500 rpm, 4°C), and supernatants were collected, divided, and stored at −80°C. Serum levels of CytC, caspase‐3, and caspase‐9 were analyzed using ELISA kits as per the provided directions.

### 2.14 Statistical Analyses

Microsoft Excel software and SPSS 23.0 software were employed to collect and analyze data, using one‐way factorial ANOVAs when data between multiple groups were normally distributed and consistent with a chi‐square test, with further pairwise comparisons with Tukey's test. When data were normally distributed but inconsistent with the chi‐square test, independent sample *t*‐tests or Dunnett's T3 tests were instead used. Skewed data were assessed with a Kruskal–Wallis H‐test. *α* = 0.05 and *p* < 0.05 were regarded as significant, and data were provided as means ± standard deviation ($\bar{x}$ ± s).

## 3 Results

### 3.1 Identification of Targets Associated With AS‐IV and CIRI

**FIGURE 3:**
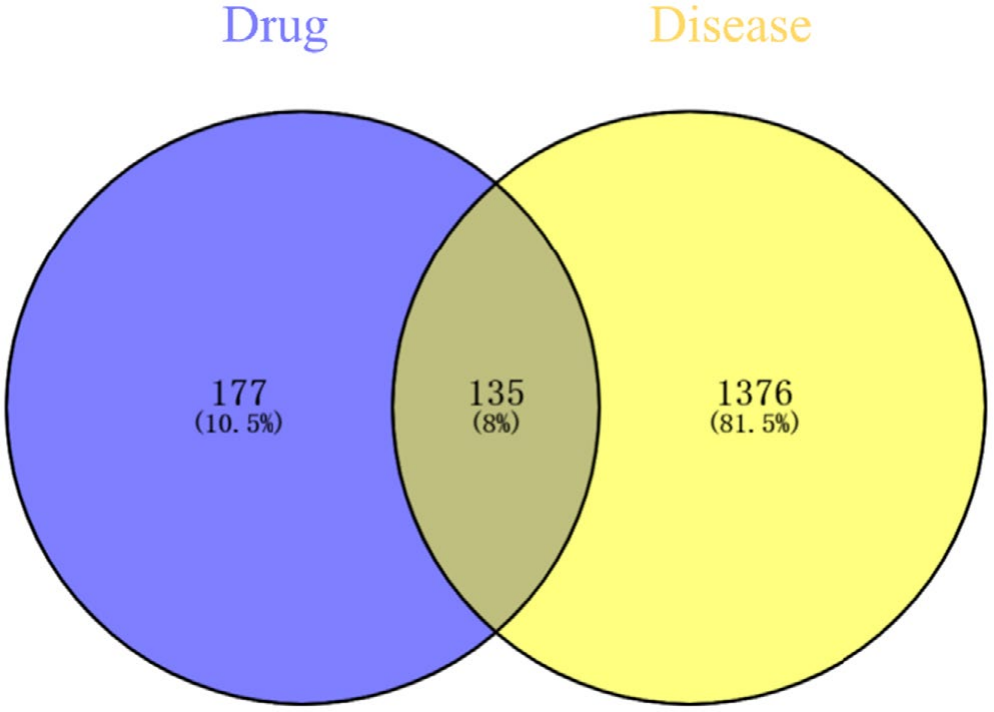
Venn diagram of the unique and overlapping targets associated with AS‐IV and CIRI.

A total of 312 unique AS‐IV targets were identified from the Swiss Target Prediction (28 targets) and PharmMapper (299 targets) databases. Moreover, 13 and 1505 CIRI‐related targets were obtained from the OMIM and Gene‐Cards databases, respectively, for 1511 unique targets. An overlap analysis identified 135 common targets shared by the AS‐IV (312 targets) and CIRI (1511 targets) datasets (Figure 3).

### 3.2 Construction and Analysis of a PPI Network

**FIGURE 4:**
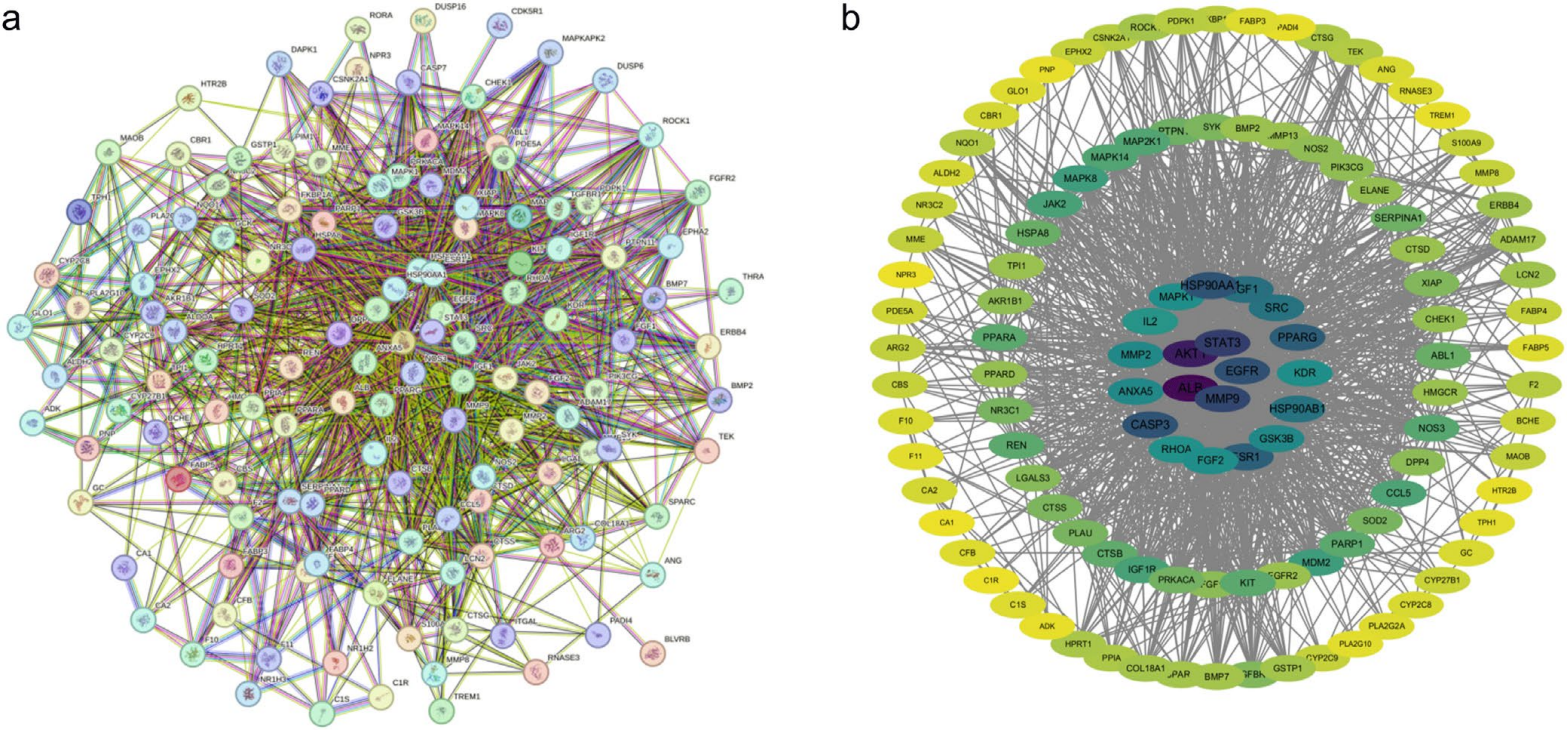
PPI network for the overlapping targets of AS‐IV and CIRI. (a) STRING database PPI network; (b) Cytoscape topological analysis of the PPI network.

Potential AS‐IV targets for CIRI treatment were analyzed using the STRING database to construct a PPI network, visualized with Cytoscape 3.10.1 (Figure 4). Network topology analysis identified 48 core target proteins, with top‐ranked targets including ALB, AKT1, STAT3, MMP9, EGFR, HSP90AA1, CASP3, PPARG, ESR1, IGF1, SRC, HSP90AB1, MMP2, GSK3B, ANXA5, FGF2, KDR, MAPK1, and MAPK8.

### 3.3 Functional Enrichment Analyses

**FIGURE 5:**
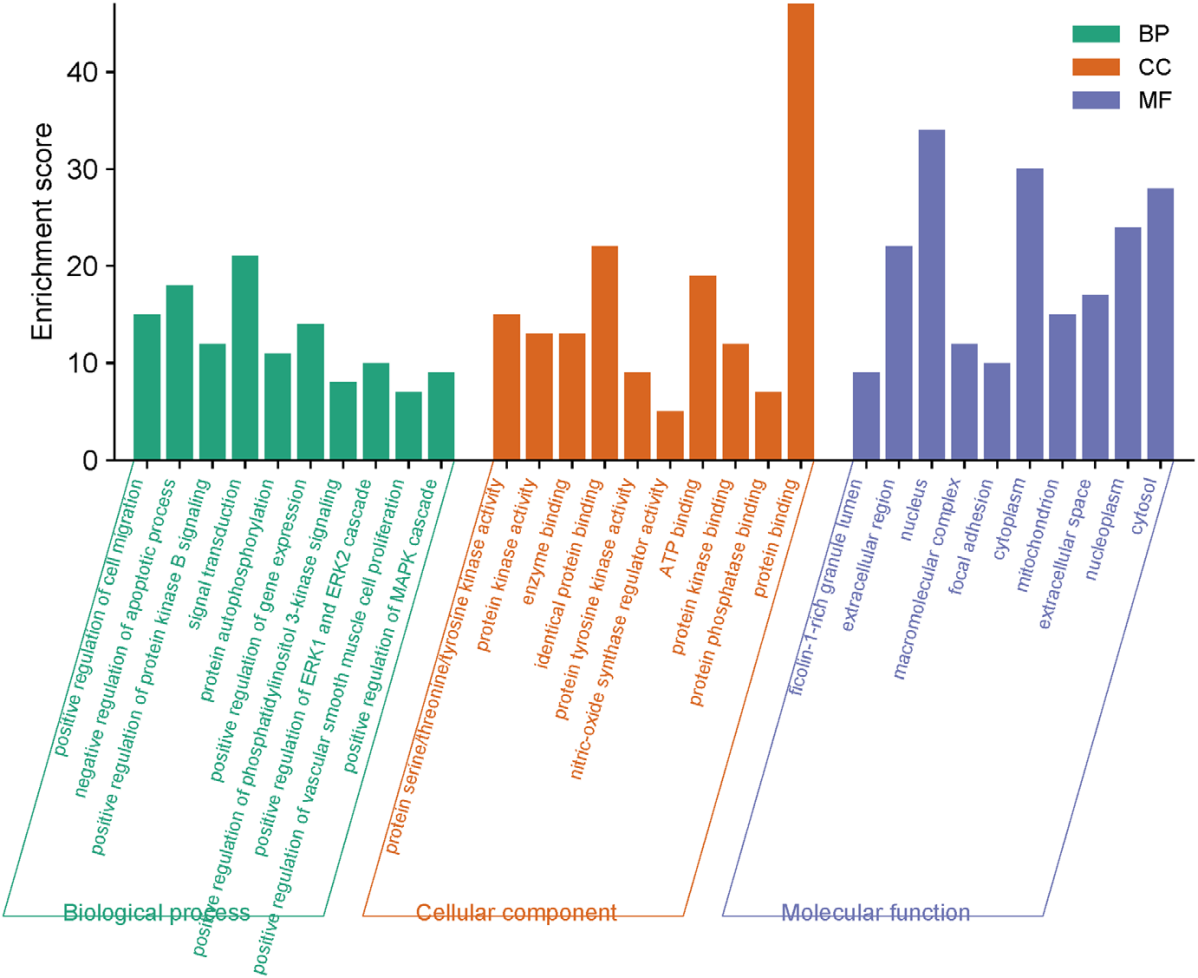
GO enrichment analysis results for key targets.

**FIGURE 6:**
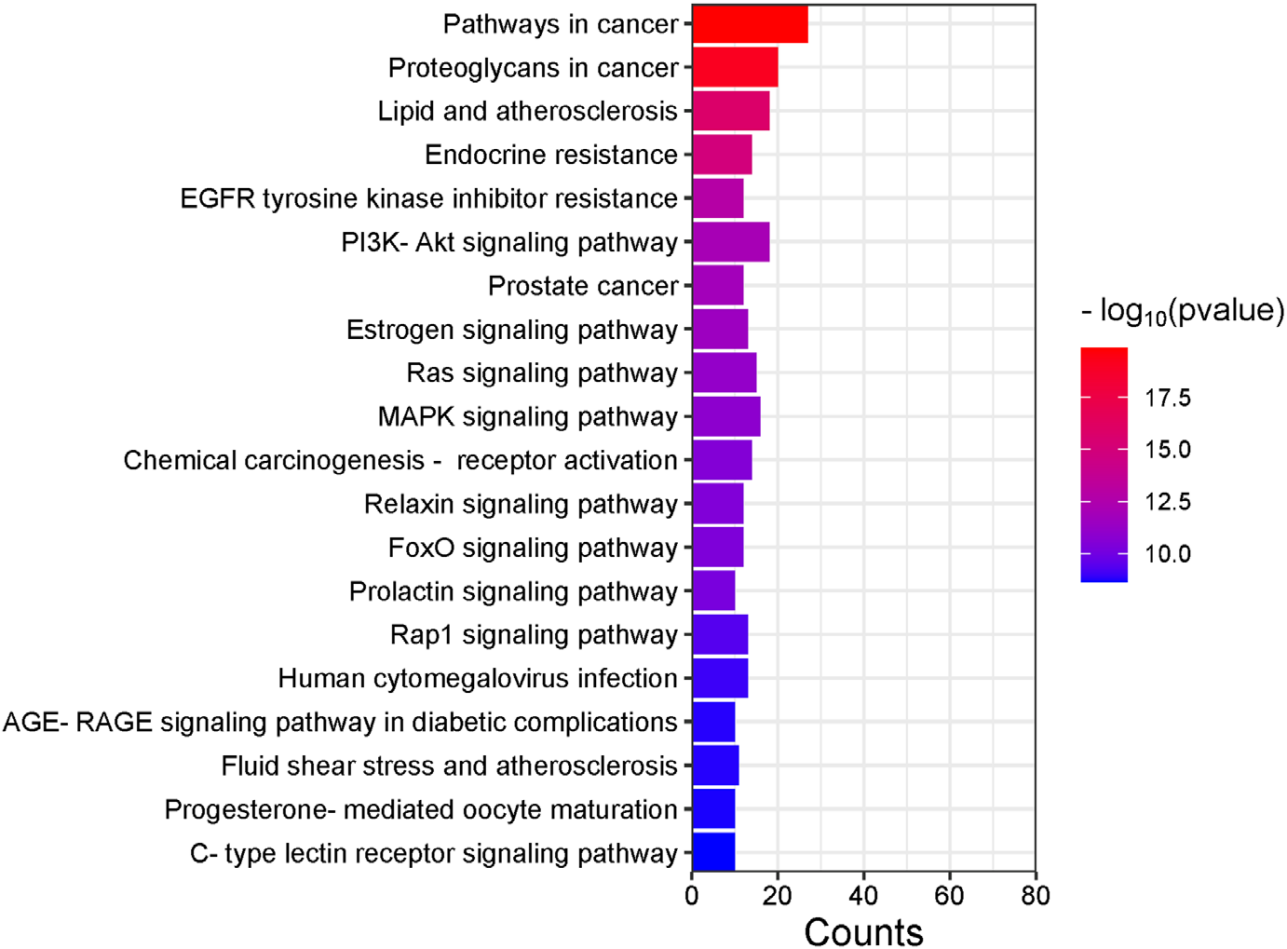
The top 20 most enriched KEGG pathways.

Enrichment analyses using the DAVID database identified 319 BP, 36 MF, 40 CC terms, and 136 KEGG pathways associated with key targets (*p* < 0.05) (Figure 5). Identified BP terms included positive regulating cell migration, negative regulation of apoptotic processes, positive regulation of protein kinase B signaling, signal transduction, and protein autophosphorylation. CC terms included protein serine/threonine/tyrosine kinase activity, protein kinase activity, and enzyme binding. MF terms included ficolin‐1‐rich granular lumen, extracellular region, nucleus, macromolecular complex, and focal adhesion. The top 20 most strongly enriched KEGG pathways are presented in Figure 6 and included the pathways in cancer, proteoglycans in cancer, lipid and atherosclerosis, endocrine resistance, EGFR tyrosine kinase inhibitor resistance, PI3K/Akt signaling, prostate cancer, estrogen signaling, Ras signaling, and MAPK signaling pathways.

### 3.4 Molecular Docking Analyses

#### 3.4.1 Molecular Docking of Key Protein Targets in PPI Networks

**FIGURE 7:**
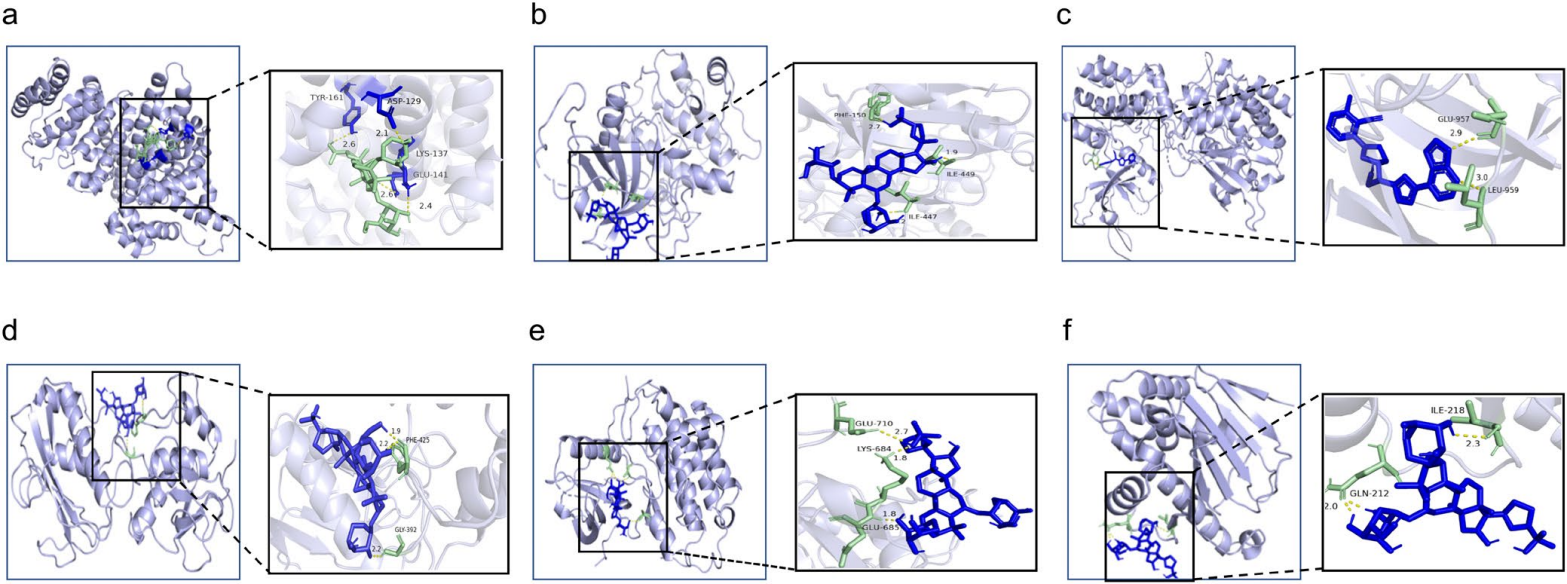
Molecular docking results for key protein targets in PPI networks. (a) ALB (PDB ID: 7D6J); (b) AKT1 (PDB ID: 4 GV1); (c) STAT3 (PDB ID: 6SM8); (d) MM9 (PDB ID: 1GKC); (e) EGFR (PDB ID: 2GS2); (f) HSP90AA19 (PDB ID: 30Oi).

Initial molecular docking analyses were performed to examine potential binding between AS‐IV and top targets from the PPI network. All binding energy values were below −5.0 kJ·mol^−1^, indicating strong binding potential (Figure 7, Table 2).

**TABLE 2:** Molecular binding energy between AS‐IV and key CIRI‐related target proteins.

Targets	PDB ID	Molecular binding energy (kJ·mol^−1^)
ALB	7D6J	−21.9
AKT1	4GV1	−14.8
STAT3	6SM8	−13.5
MM9	1GKC	−12.5
EGFR	2GS2	−12.4
HSP90AA19	30Oi	−18.81

#### 3.4.2 Molecular Docking Analyses of JNK/Bid Signaling Pathway Proteins

**FIGURE 8:**
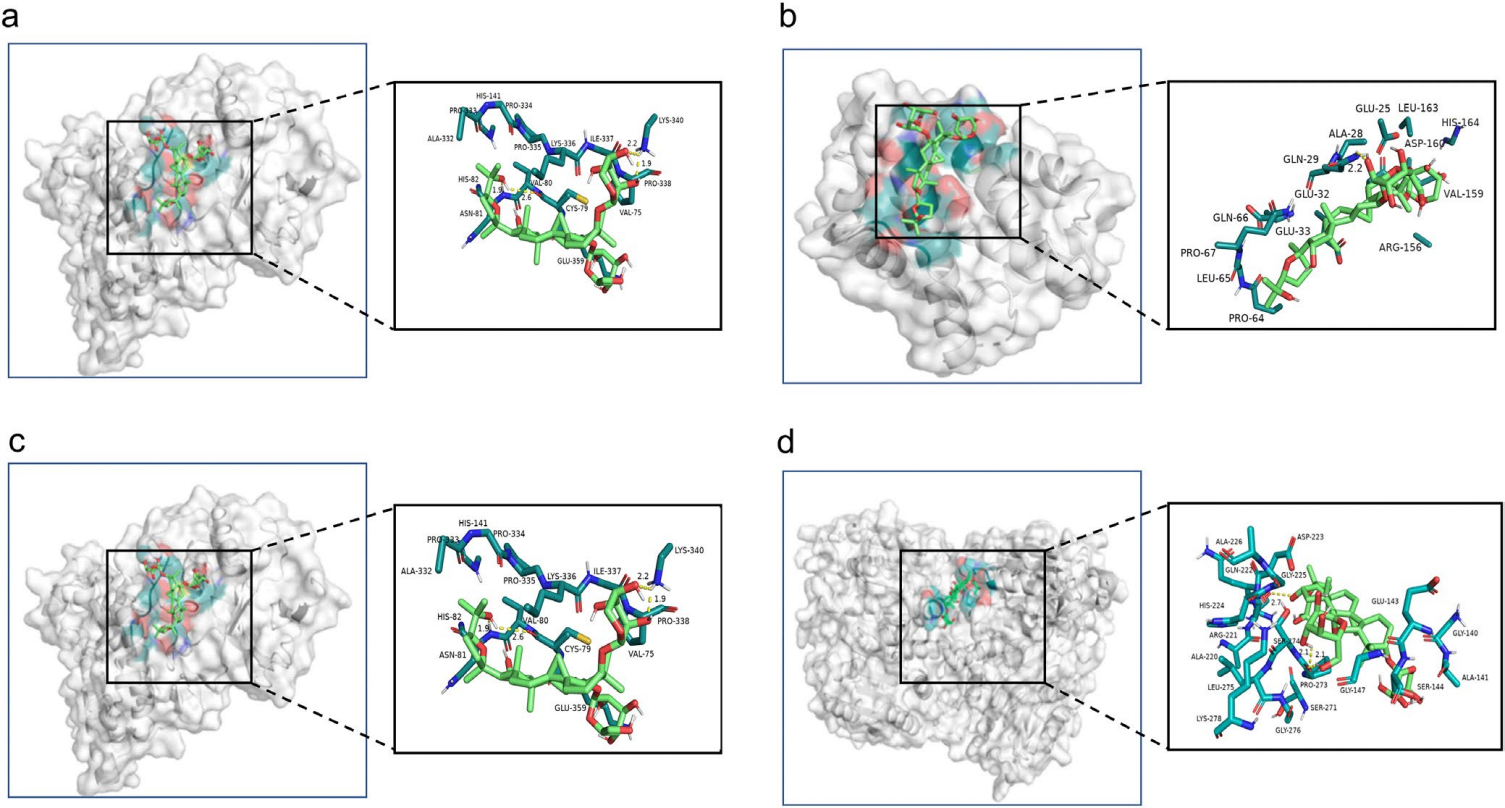
Molecular docking results for key protein targets in JNK/Bid signaling pathway. (a) JNK (PDB ID: 4QTD); (b) Bid (PDB ID: 7M5A); (c) Caspase‐3 (PDB ID: 3GJQ); (d) Caspase‐9 (PDB ID: 2AR9).

Based on the above network pharmacology results and prior publications, AS‐IV was speculated to affect CIRI via the JNK/Bid signaling pathway, which belongs to the MAPK pathway. As such, molecular docking analyses were further used to test for potential interactions between AS‐IV and key targets of the JNK/Bid pathway to clarify whether this axis may underlie the protective benefits of this compound. AS‐IV docked with JNK, Bid, caspase‐3, and caspase‐9, exhibiting binding energy levels below −5.0 kJ·mol^−1^ (Figure 8, Table 3).

**TABLE 3:** Molecular binding energy between AS‐IV and key JNK/Bid pathway proteins.

Targets	PDB ID	Molecular binding energy (kJ·mol^−1^)
JNK	4QTD	−21.2
Bid	7M5A	−13.8
Caspase‐3	3GJQ	−23.3
Caspase‐9	2AR9	−12.3

### 3.5 The Impact of AS‐IV on Neurological Functionality

**FIGURE 9:**
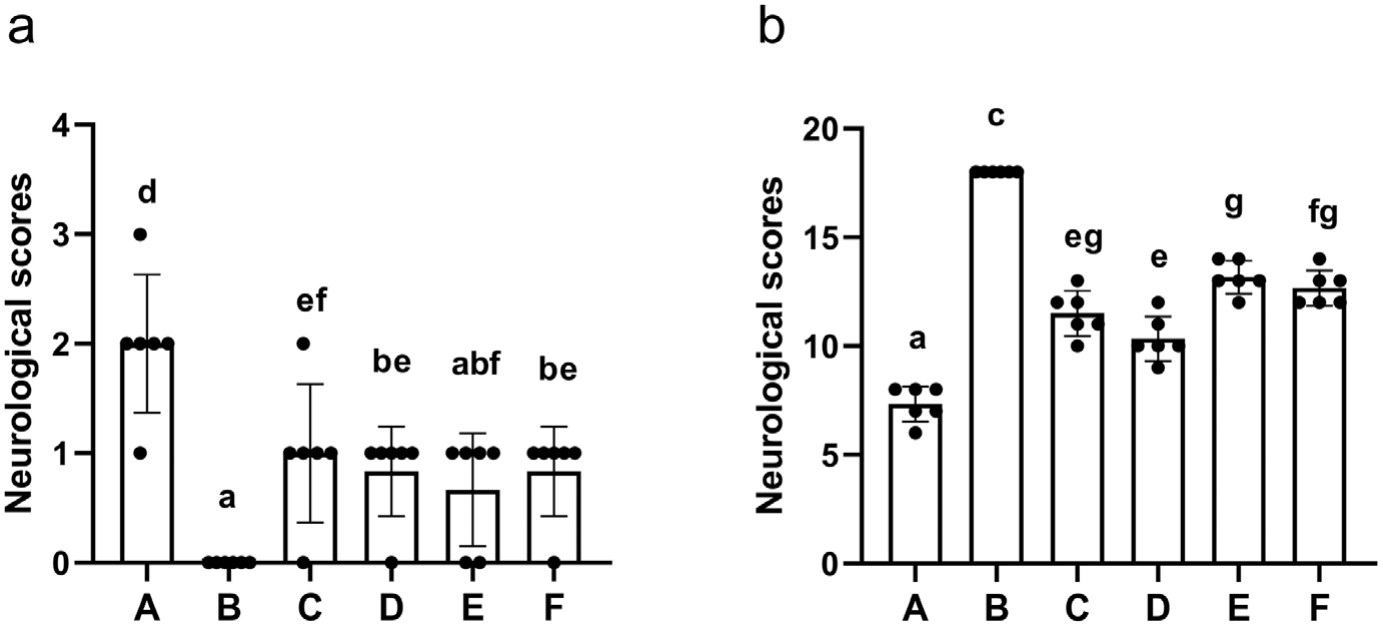
The impact of AS‐IV on the neurological function scores ($\bar{x}$ ± s, *n* = 6). (a) Zea Longa neurobehavioral scoring method; (b) Garcia JH neurobehavioral scoring method; A, MCAO/R group; B, Sham group; C, AS‐IV group; D, SP600125 group; E, AS‐IV + SP600125 group; F, NBP group. Significant differences are marked with different letters (*p* < 0.05 or *p* < 0.01).

In the Zea Longa neurobehavioral scoring method, no clear neurological deficits were observed for rats in the sham control group, whereas those in the MCAO/R group showed significantly higher neurological scores (*p* < 0.01). Relative to the MCAO/R group, significant decreases in neurological function scores were observed in the AS‐IV, SP600125, AS‐IV + SP600125, and NBP groups after treatment for 7 days (*p* < 0.05 or *p* < 0.01) (Figure 9a). In the Garcia JH scoring method, MCAO/R rats showed significantly lower neurological scores relative to the sham group (*p* < 0.01). Treatment significantly improved these scores (*p* < 0.01) (Figure 9b).

### 3.6 The Impact of AS‐IV on Cerebral Infarct Volume

**FIGURE 10:**
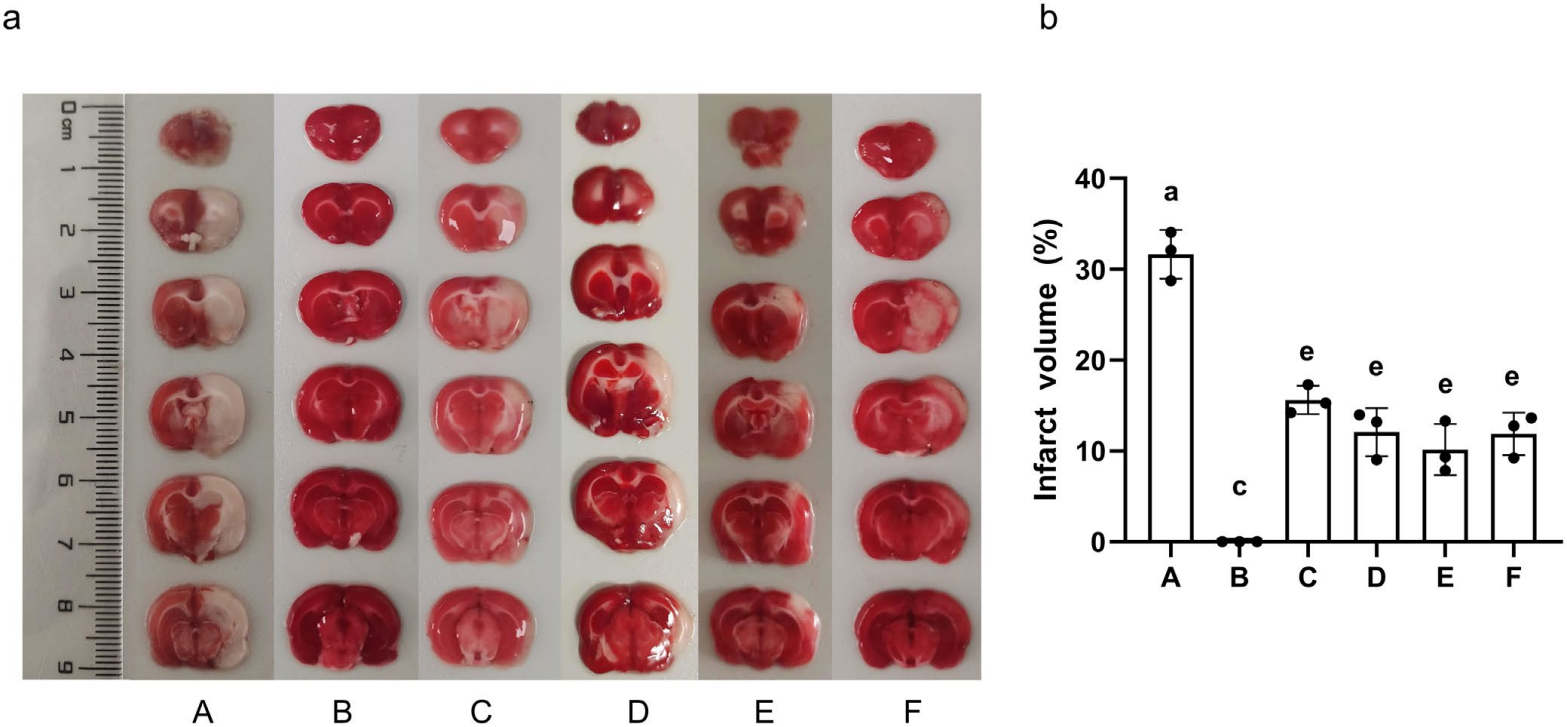
The effect of AS‐IV on cerebral infarction volume. (a) Representative TTC staining images; (b) Percentage of cerebral infarction volume ($\bar{x}$ ± s, *n* = 3); A, MCAO/R group; B, Sham group; C, AS‐IV group; D, SP600125 group; E, AS‐IV + SP600125 group; F, NBP group. Significant differences are marked with different letters (*p* < 0.05 or *p* < 0.01).

TTC staining revealed red regions in normal tissues, while ischemic tissues showed pale staining (Figure 10a). Relative to the sham group, MCAO/R rats presented with clear infarcts and a significant rise in infarct volume (*p* < 0.01) (Figure 10b). The AS‐IV, SP600125, AS‐IV + SP600125, and NBP groups showed significantly reduced infarct volumes compared to the MCAO/R group (*p* < 0.01).

### 3.7 AS‐IV Protects Against Neuronal Damage in the Brain Tissue

**FIGURE 11:**
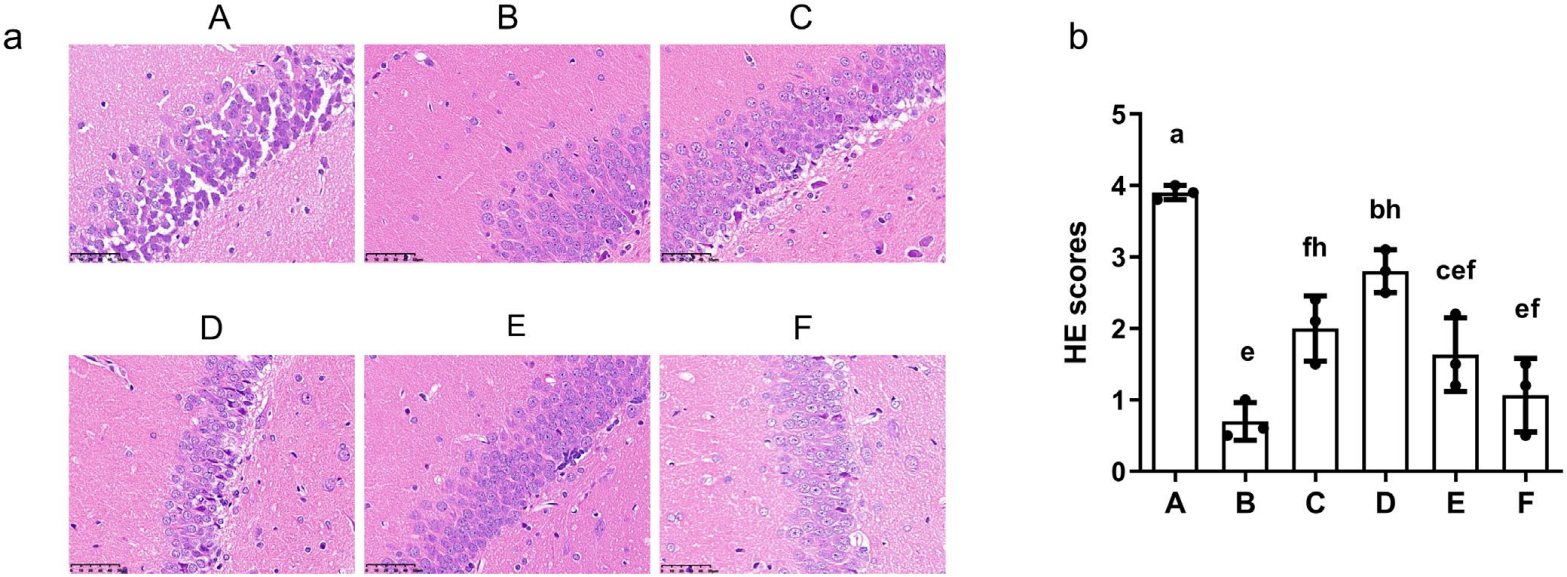
The effects of AS‐IV on pathological damage in the brain. (a) Representative H&E staining images (400×: 50 μm); (b) Semi‐quantitative H&E staining scores ($\bar{x}$ ± s, *n* = 3); A, MCAO/R group; B, Sham group; C, AS‐IV group; D, SP600125 group; E, AS‐IV + SP600125 group; F, NBP group. Significant differences are marked with different letters (*p* < 0.05 or *p* < 0.01).

Sham group brains showed structurally normal hippocampal neurons with no detectable damage. The MCAO/R group showed necrotic and atrophied neurons, tissue damage, enlarged gaps, and inflammatory infiltration (Figure 11a). AS‐IV, SP600125, AS‐IV + SP600125, and NBP treatments significantly reduced tissue damage compared to the MCAO/R group (*p* < 0.05 or *p* < 0.01). The severity of tissue damage followed this order: NBP group < AS‐IV + SP600125 group < AS‐IV group < SP600125 group (Figure 11b).

### 3.8 The Effect of AS‐IV on the Apoptosis in the Brain Tissue

**FIGURE 12:**
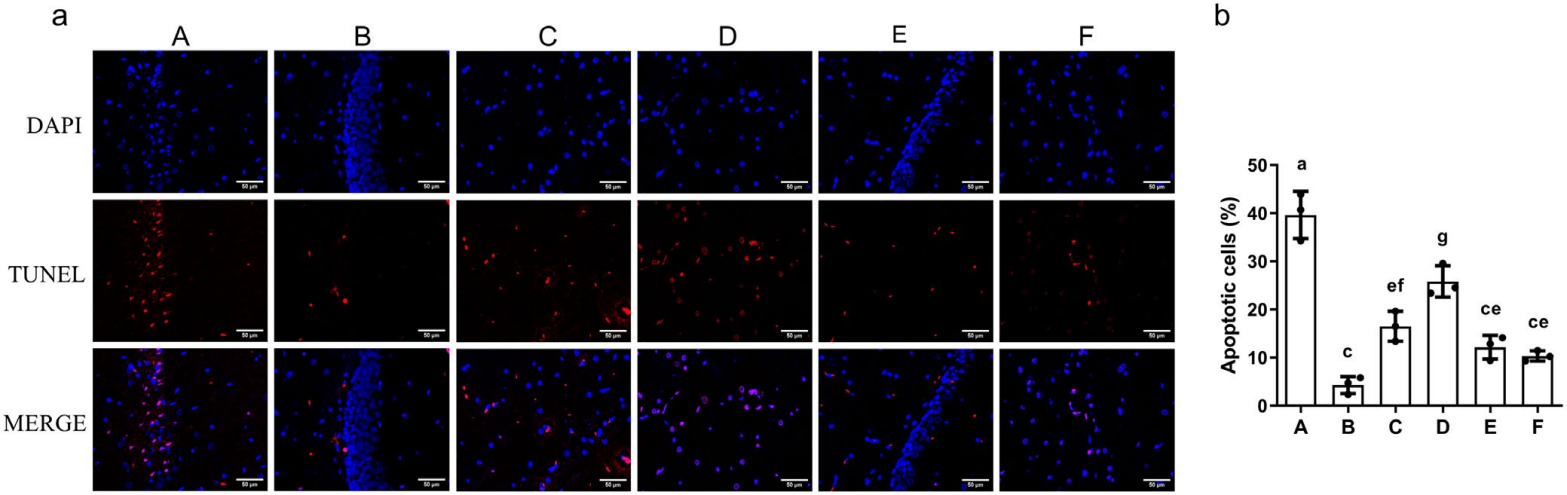
The impact of AS‐IV on apoptosis in the brain. (a) Representative TUNEL staining images (200×: 50 μm); (b) Apoptosis rate ($\bar{x}$ ± s, *n* = 3); A, MCAO/R group; B, Sham group; C, AS‐IV group; D, SP600125 group; E, AS‐IV + SP600125 group; F, NBP group. Significant differences are marked with different letters (*p* < 0.05 or *p* < 0.01).

Hippocampal apoptosis rates were significantly elevated in the MCAO/R group compared to sham control (*p* < 0.01) but were significantly reduced in the AS‐IV, SP600125, AS‐IV + SP600125, and NBP groups (*p* < 0.01) (Figure 12).

### 3.9 The Effect of AS‐IV on the Related Positive Expression in the Brain Tissue

**FIGURE 13:**
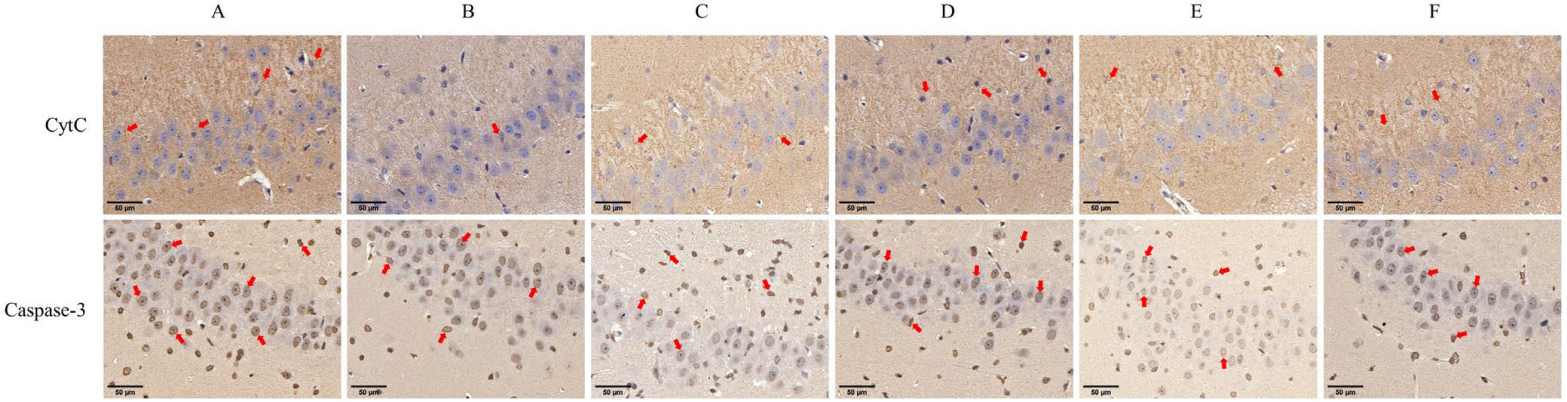
The impact of AS‐IV on CytC and caspase‐3 levels in the brain (200×: 50 μm, *n* = 3). A, MCAO/R group; B, Sham group; C, AS‐IV group; D, SP600125 group; E, AS‐IV + SP600125 group; F, NBP group.

Immunohistochemical staining revealed that the neurons of rats in the sham group were larger, with lightly stained nuclei that were round and exhibited a visible nucleolus (Figure 13). Neurons in the MCAO/R group showed enlarged morphology, round nuclei, and increased CytC and caspase‐3 expression. AS‐IV and SP600125 treatments reduced CytC and caspase‐3 expression, with AS‐IV showing a more pronounced effect. Reductions in the AS‐IV + SP600125 group were greater than those in either of the single‐agent treatment groups. The NBP group showed low positive CytC and caspase‐3 expression. AS‐IV treatment thus effectively reduced CytC and caspase‐3 levels in the brains of treated rats.

### 3.10 AS‐IV Affects Apoptosis‐Associated Protein Levels in the Brain Tissue

**FIGURE 14:**
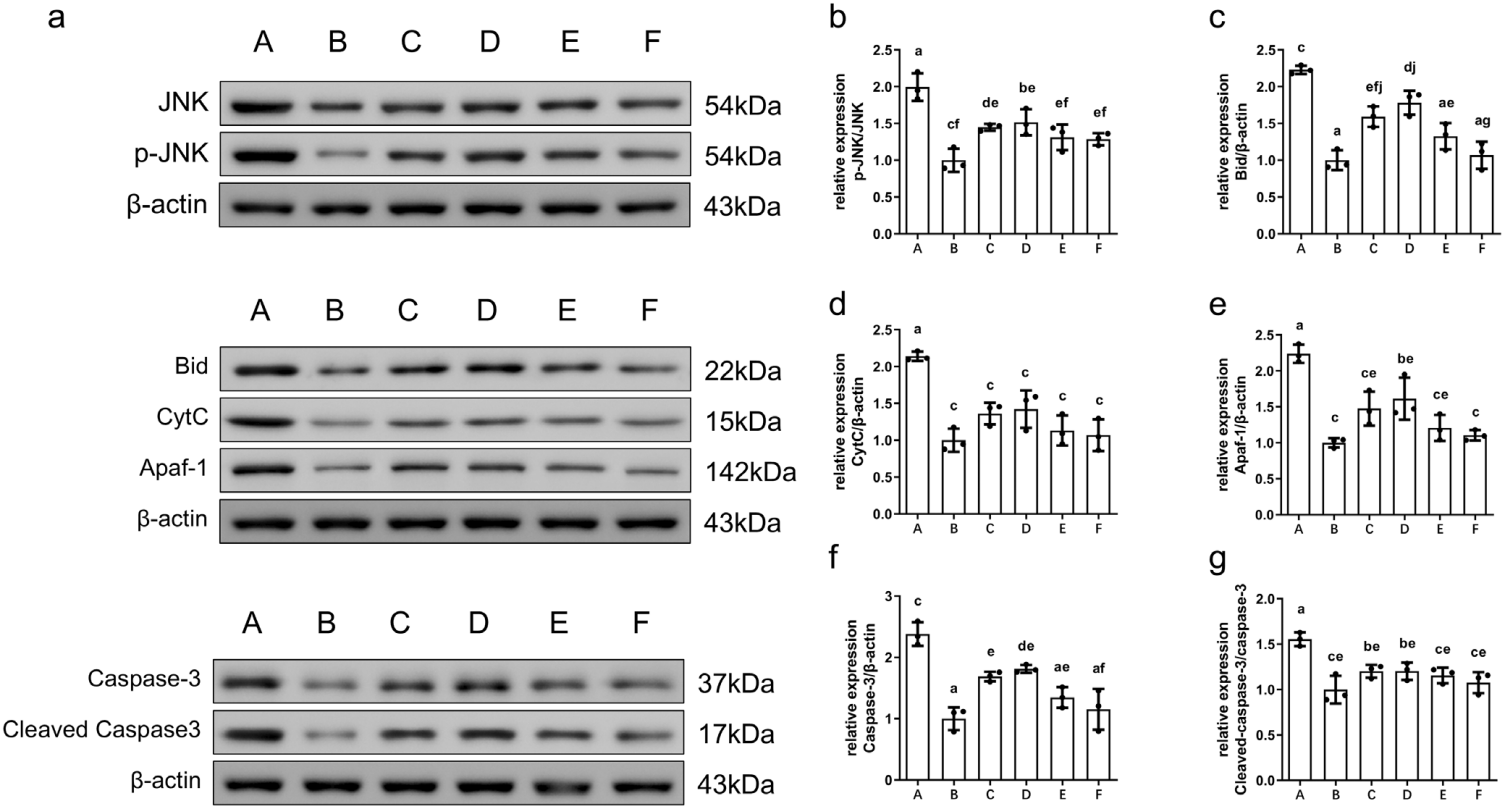
The impact of AS‐IV on apoptosis‐associated protein levels in the brain ($\bar{x}$ ± s, *n* = 3). (a) Representative Western blotting images; (b) P‐JNK/JNK levels; (c) Bid levels; (d) CytC levels; (e) Apaf‐1 levels; (f) Caspase‐3 levels; (g) Cleaved caspase‐3/caspase‐3 levels; A, MCAO/R group; B, Sham group; C, AS‐IV group; D, SP600125 group; E, AS‐IV + SP600125 group; F, NBP group. Significant differences are marked with different letters (*p* < 0.05 or *p* < 0.01).

MCAO/R rats showed significantly elevated p‐JNK, Bid, CytC, Apaf‐1, caspase‐3, and cleaved caspase‐3 levels relative to the sham group (*p* < 0.01). These levels were significantly reduced in the AS‐IV, SP600125, AS‐IV + SP600125, and NBP groups (*p* < 0.05 or *p* < 0.01) (Figure 14).

### 3.11 AS‐IV Alters Apoptosis‐Associated mRNA Expression in the Brain Tissue

**FIGURE 15:**
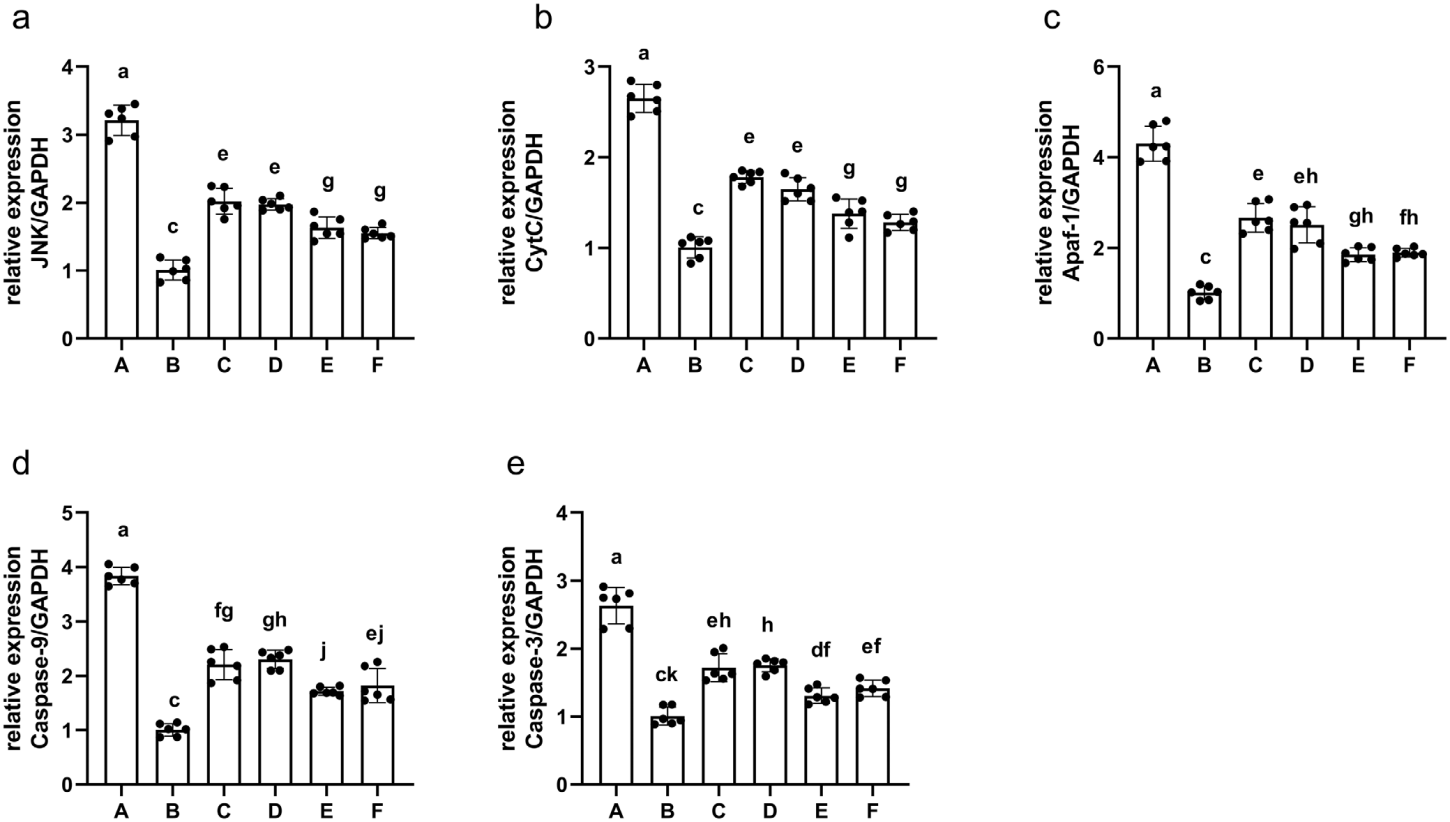
The impact of AS‐IV on apoptosis‐associated mRNA levels in the brain ($\bar{x}$ ± s, *n* = 3). (a) JNK levels; (b) CytC levels; (c) Apaf‐1 levels; (d) Caspase‐9 levels; (e) Caspase‐3 levels; A, MCAO/R group; B, Sham group; C, AS‐IV group; D, SP600125 group; E, AS‐IV + SP600125 group; F, NBP group. Significant differences are marked with different letters (*p* < 0.05 or *p* < 0.01).

Key gene expression was analyzed via qPCR in rat brain samples (Figure 15). Relative to the sham group, increased JNK, CytC, Apaf‐1, caspase‐3, and caspase‐9 expression at the mRNA level was evident in the MCAO/R group (*p* < 0.01), while these levels were significantly reduced in the AS‐IV, SP600125, AS‐IV + SP600125, and NBP groups (*p* < 0.01). These findings suggested that AS‐IV can inhibit JNK expression and have a potential anti‐apoptotic effect on rat brains.

### 3.12 AS‐IV Affects Serum Levels of Apoptosis‐Related Factors

**FIGURE 16:**
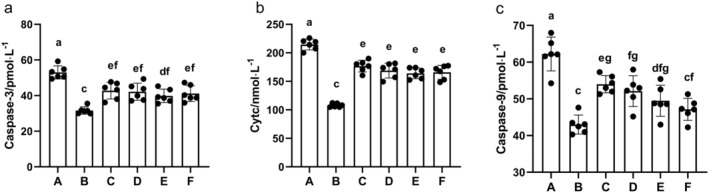
The impact of AS‐IV on serum levels of apoptosis‐related factors ($\bar{x}$ ± s, *n* = 6). (a) Caspase‐3; (b) CytC; (c) Caspase‐9; A, MCAO/R group; B, Sham group; C, AS‐IV group; D, SP600125 group; E, AS‐IV + SP600125 group; F, NBP group. Significant differences are marked with different letters (*p* < 0.05 or *p* < 0.01).

Serum levels of CytC, caspase‐3, and caspase‐9 were significantly elevated in MCAO/R rats compared to the sham group (*p* < 0.01). These levels were significantly reduced in the AS‐IV, SP600125, AS‐IV + SP600125, and NBP groups (*p* < 0.01) (Figure 16).

## 4 Discussion

IS is associated with high rates of incidence, disability, and mortality, posing significant challenges to public health and increasing healthcare costs. CIRI occurs following reperfusion through mechanisms such as oxidative stress, inflammation, nitric oxide injury, excitatory amino acid toxicity, Ca^2+^ overload, and apoptosis [29, 30]. Despite extensive research, effective interventional strategies for CIRI remain limited, challenging clinical practice. Approaches to preventing or combatting CIRI are thus a focus of research aimed at improving IS patient outcomes. Further studies on IS pathology and mechanisms may facilitate the development of more effective treatments.

Here, a network pharmacology approach was employed to identify 312 AS‐IV‐related targets, and 48 key targets of AS‐IV predicted to treat CIRI. PPI topology analyses identified ALB, AKT1, STAT3, MMP9, and EGFR as potential core targets for the AS‐IV‐mediated treatment of CIRI. AKT1 exhibits anti‐apoptotic effects by inactivating pro‐apoptotic Bad, suppressing Bax translocation and CytC release [31]. STAT3, a downstream target of JAK, regulates proliferation, inflammation, and apoptosis through the JAK/STAT3 pathway. CIRI can induce JAK2 and STAT3 phosphorylation and thereby drive neuronal apoptosis [32]. An elevated p‐STAT3/STAT3 ratio favors the M2 differentiation of brain microglia, thereby helping to protect against neuroinflammation [32]. EGFR is involved in diverse cellular responses that include enhanced proliferation and the suppression of apoptosis, having important effects related to β‐Catenin, which has been shown to help protect against myocardial ischemia–reperfusion injury. The specific role of EGFR in CIRI, however, remains uncertain [33]. Functional enrichment analyses suggested that AS‐IV mitigates neuronal damage and oxidative stress in CIRI by regulating negative apoptosis, signal transduction, protein phosphorylation, and pathways such as PI3K‐Akt, Ras, MAPK, FoxO, Fluid shear stress, and atherosclerosis. Consistent with the prediction that the protective effects of AS‐IV against CIRI may be associated with MAPK signaling, molecular docking studies demonstrated the ability of AS‐IV to bind with key JNK/Bid pathway targets.

CIRI is characterized by heightened apoptotic [34, 35], which is central to tissue damage following ischemic events. Here, severe brain damage was evident in MCAO/R rats, with high rates of apoptotic induction. In CIRI, Bcl‐2 family proteins regulate mitochondrial membrane permeability [36] and mediate apoptosis via the endogenous pathway. These proteins are classified into pro‐apoptotic factors (Bax, Bak), anti‐apoptotic factors (Bcl‐2, Bcl‐XL, Bcl‐W), and pro‐apoptotic BH3‐only proteins (Bid). The relative levels of these different proteins ultimately shape cellular survival [37]. Bax can promote greater outer mitochondrial membrane permeability, whereas Bcl‐2 has the opposite effect by suppressing pro‐apoptotic and BH3‐only protein activation [38]. A shift in mitochondrial membrane permeability is key to the apoptotic process, mediating the release of factors such as CytC from mitochondria to cytoplasm [39, 40], which can further trigger this apoptotic cell death pathway [41]. The binding of CytC to Apaf‐1 can promote caspase‐3/9 activation, with both of these proteins serving as key mediators of neuronal apoptosis, and both being activated downstream of the exogenous and endogenous apoptotic pathways [42], resulting in irreversible apoptotic induction. Caspase‐8/9/10 are considered apoptosis‐promoting type I caspases, whereas caspase‐3/6/7 are executor type II caspases, and partial caspases serve as regulators of inflammatory activity [43]. Mitochondria represent the major source and target of oxidative stress, with high ROS production following CIRI coinciding with the mitochondria dysfunction in injured cells, effectively inducing apoptotic death [44].

Apoptosis is closely associated with JNK signaling and the process of CIRI. Following CIRI, the JNK signaling pathway is activated to modulate apoptosis‐related protein expression, including members of the Bcl‐2 family. Specifically, it suppresses Bcl‐2 expression while activating BH3‐only proteins with pro‐apoptotic activity, thereby inducing mitochondrial‐dependent apoptosis. Bid is a BH3‐only protein that favors Bax and Bak channel formation and damage to the mitochondrial membrane [41, 45, 46]. Peak p‐JNK levels have been observed at 6 h following CIRI, whereas peak apoptotic death is evident after 12 h and decreases substantially following JNK inhibition [33]. JNK can further upregulate pro‐apoptotic factors such as p53, FasL, and BimEL by activating C‐Jun, JunB, JunD, and other transcription factors [33]. Inhibiting JNK can suppress apoptosis by preventing mitochondrial Bim and Bax translocation, CytC and Smac release, and caspase‐3/9 activation [47]. SP600125 (C_14_H_8_N_2_O) is a reversible, selective JNK inhibitor capable of crossing the BBB. It can inhibit JNK phosphorylation by reversibly competing for ATP, thus inhibiting the JNK signal transduction pathway [48, 49]. SP600125 can prevent neuronal apoptosis, neuroinflammation, and impaired memory function [50, 51]. Here, SP600125 was employed for specific JNK inhibition to explore the ability of AS‐IV to protect against post‐CIRI apoptosis through the regulation of JNK/Bid signaling. It was reported that HSC70 or eEF1A1 knockdown increases phosphorylated JNK, phosphorylation of c‐JUN, cleaved caspase‐9, and cleaved caspase‐3 expression in the background of ischemic stroke, which could be rescued by SP600125 [52]. These indicated that inhibiting the JNK pathway activation can play a protective role in stroke, which is consistent with our research results.

AS‐IV, the primary saponin component of *Astragali radix*, has been shown to reduce CIRI‐induced cell damage [27, 53, 54]. AS‐IV has been demonstrated to penetrate the BBB [55] and to protect against CIRI‐induced apoptotic death by inhibiting key targets involved in the mitochondrial and death receptor pathways [56]. AS‐IV can also reportedly protect astrocytes against OGD/R‐induced damage by inhibiting JNK signaling [57]. Here, AS‐IV significantly reduced infarct volume in the brains of MCAO/R rats and mitigated neurological deficits. H&E and TUNEL staining suggested that AS‐IV could significantly suppress neuronal damage and apoptotic induction, indicating that this may be the main pathway through which it exerts its effects. A comparable efficacy was observed following treatment with JNK inhibitors. At the protein level, AS‐IV and SP600125 both significantly reduced p‐JNK levels as well as those of pro‐apoptotic proteins (CytC, Bid, Apaf‐1) and effectors (caspase‐3 and cleaved caspase‐3). AS‐IV may thus protect against neurological damage in MCAO/R rats by inhibiting JNK and downregulating downstream JNK/Bid pathway targets, suppressing apoptotic induction.

NBP, also known as apigenin, was originally isolated from celery seeds and is the first independently developed drug in China for cardiovascular diseases, frequently used to treat IS. It shows mitochondria‐protective, brain energy metabolism‐enhancing, antiplatelet, antithrombotic, antioxidant, anti‐inflammatory, neuroprotective, and cerebral blood flow and collateral circulation‐enhancing activities [58]. NBP increases post‐CIRI ATP levels, preserves mitochondrial membrane fluidity and potential, and improves mitochondrial morphology [59, 60]. NBP can also suppress the post‐CIRI release of CytC and AIF, thereby mitigating caspase‐dependent and caspase‐independent apoptotic cascades [61]. Mechanistically, NBP has been suggested to inhibit JNK activation to protect against CIRI‐associated apoptosis [62]. In this study, AS‐IV demonstrated comparable efficacy to NBP in treating MCAO/R rats.

## 5 Conclusion

In conclusion, the present study demonstrated that AS‐IV and SP600125 reduce infarct volume in MCAO/R rats, alleviating neurological deficits and brain tissue damage. AS‐IV's neuroprotective effects were associated with the JNK/Bid pathway based on network pharmacology predictions and experimental validation. Future studies at the cellular and knockout mouse levels are required to further explore this mechanism, offering a foundation for clinical AS‐IV development for CIRI treatment.

## Author Contributions

**L. Y.:** methodology, validation, visualization, funding acquisition, writing – original draft. **W. J.:** methodology, validation, visualization, funding acquisition. **D. D.** and **Y. W.:** data curation. **Q. C.** and **Y. Z.:** writing – review and editing. **H. W.:** conceptualization, funding acquisition, supervision. **Y. C.** and **Y. C.:** conceptualization, supervision. **Y. H.** and **L. Z.:** funding acquisition, supervision.

## Conflicts of Interest

The authors declare no conflicts of interest.

## Supporting information


Data S1.

Data S2.

## Data Availability

Data will be made available on request.
