# Supplementary material for: Investigation of Anti‐Apoptotic Effects and Mechanisms of Astragaloside IV in a Rat Model of Cerebral Ischemia–Reperfusion Injury
Source: CNS Neurosci Ther. 2025 Jan 7;31(1):e70209. doi: 10.1111/cns.70209 (PMC11705586; doi:10.1111/cns.70209)

Full unedited gel/blot for Figure 14a
JNK

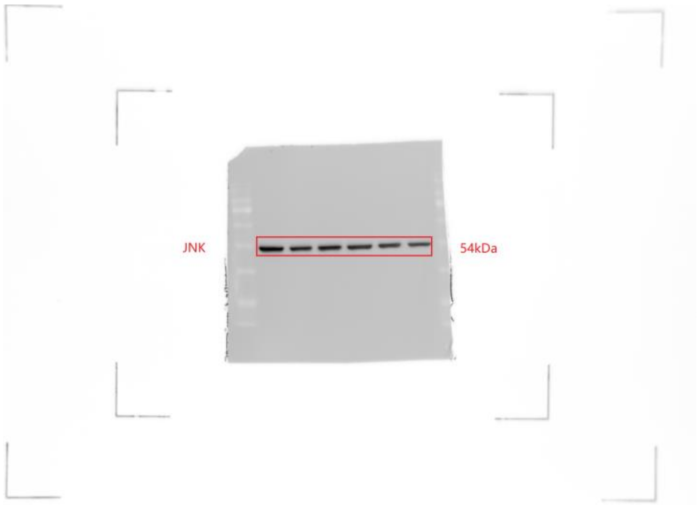

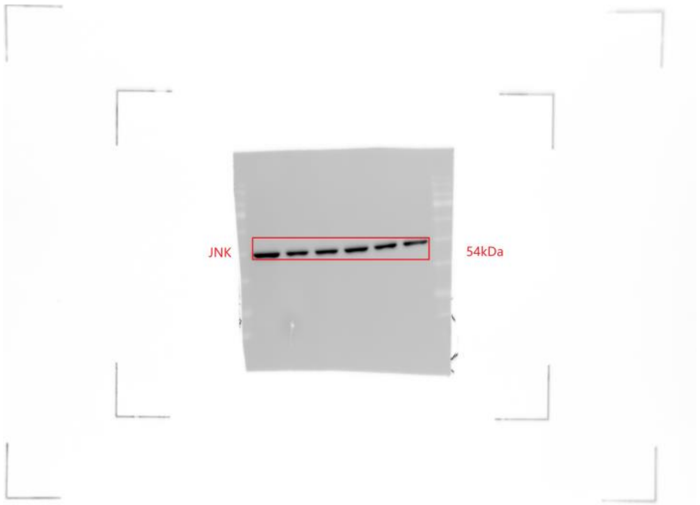

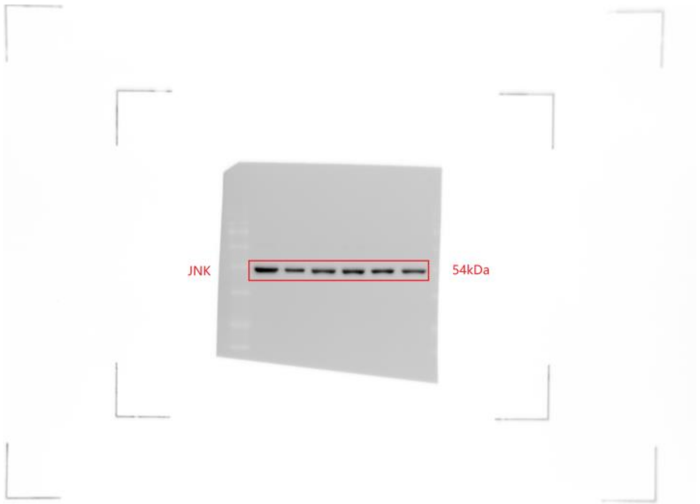

p-JNK

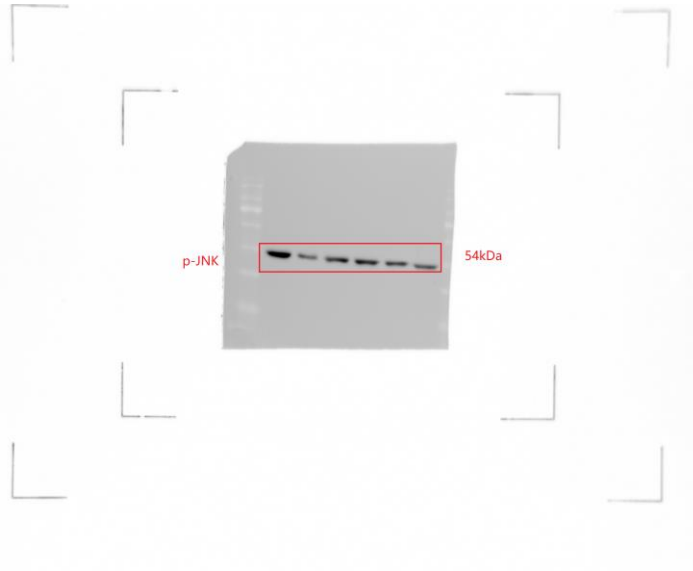

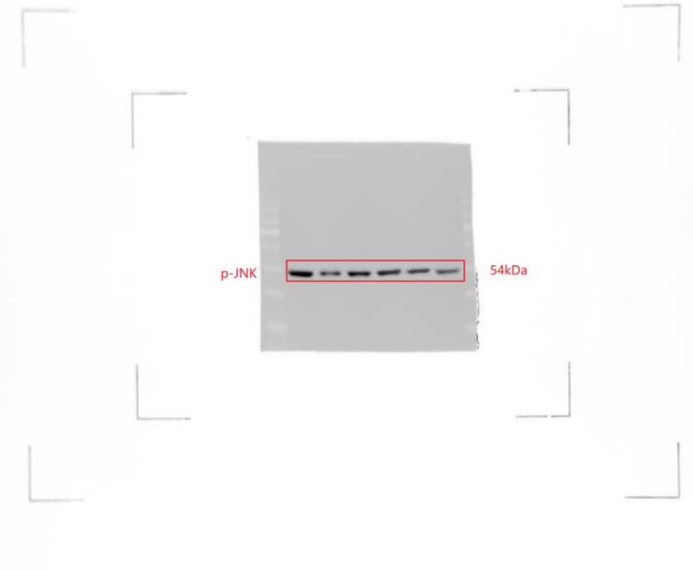

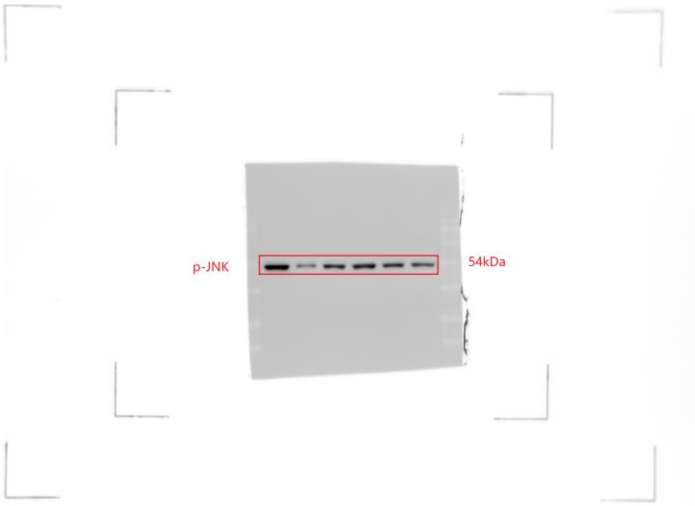

β-actin (Part 1)

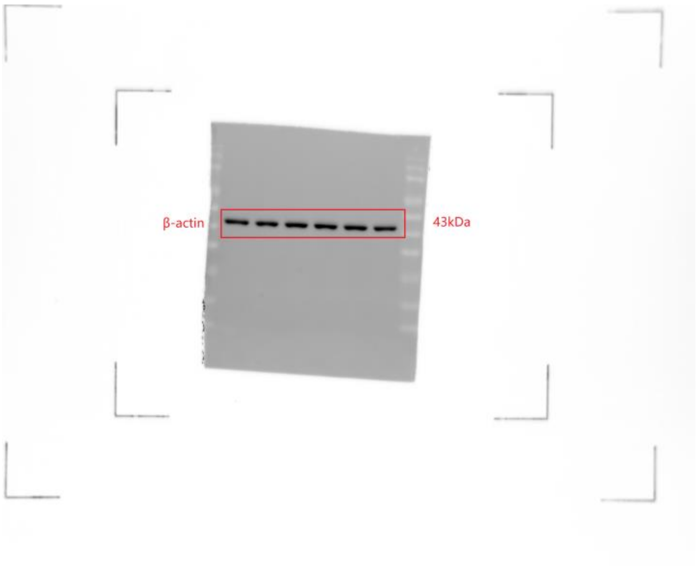

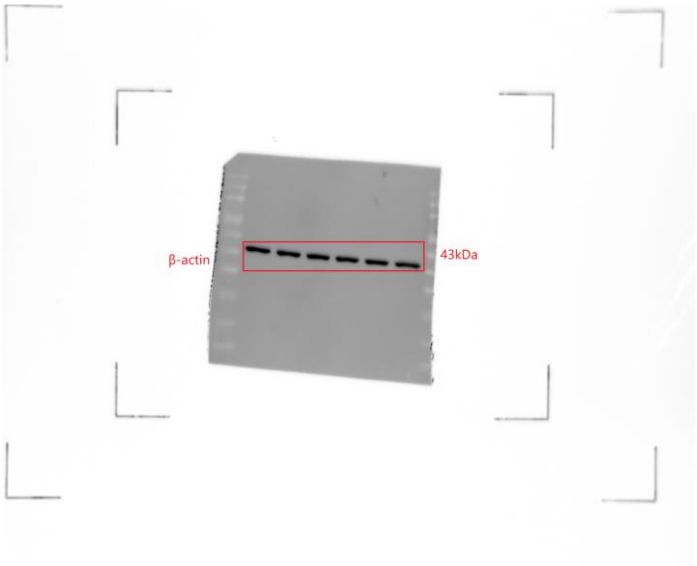

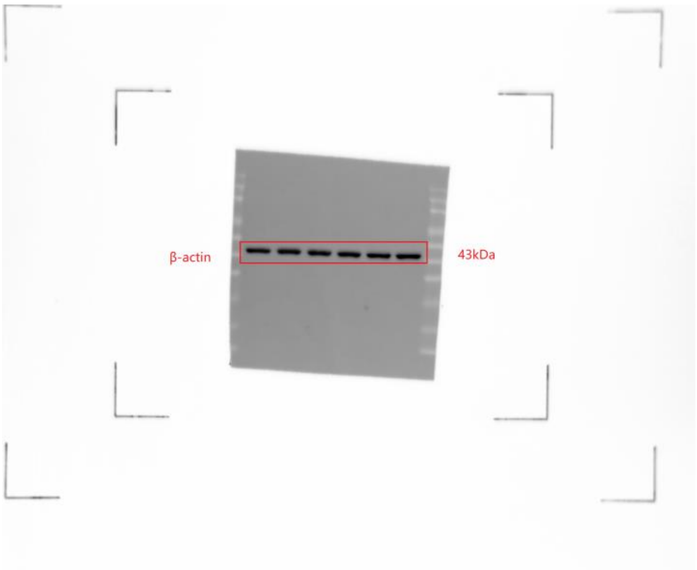

Bid

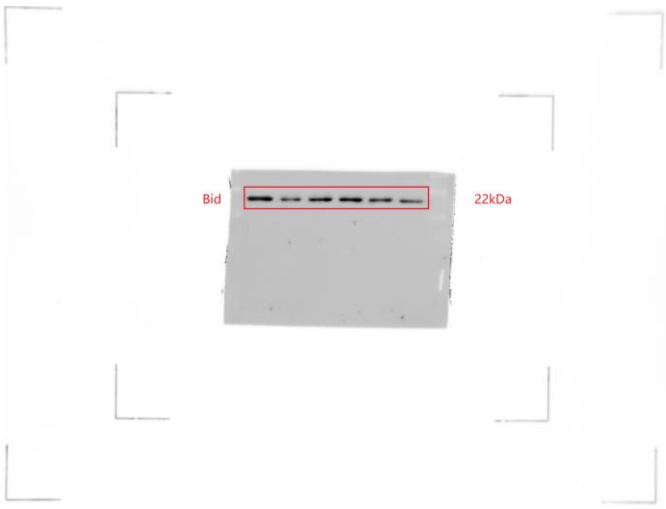

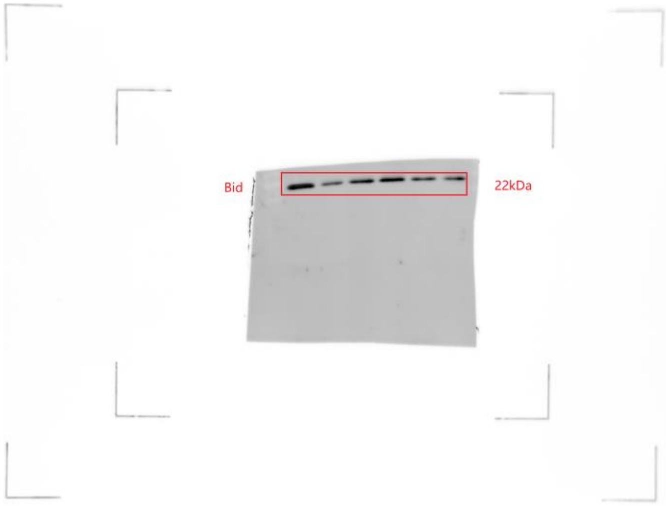

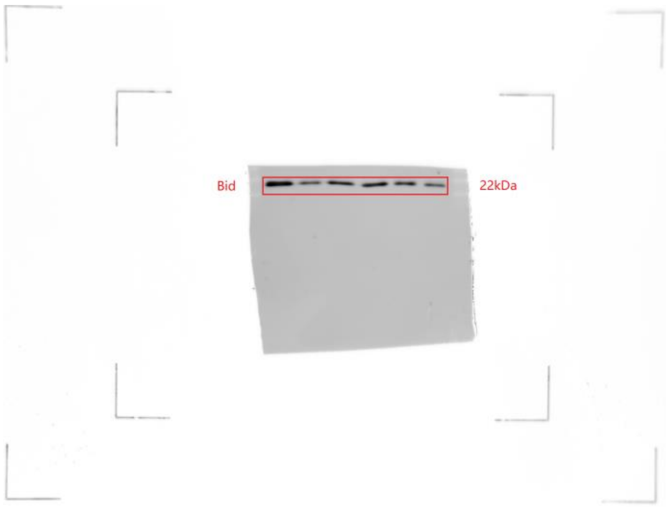

Cytc

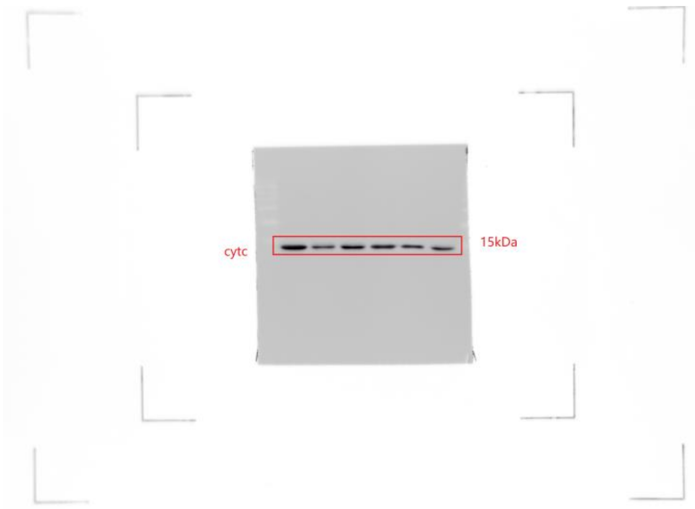

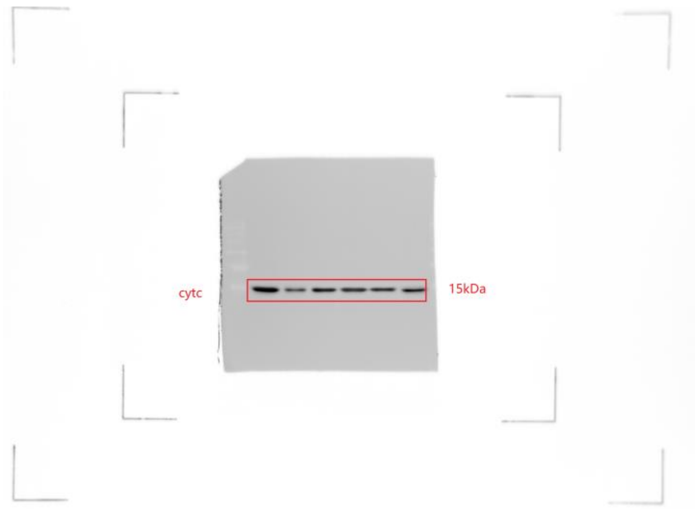

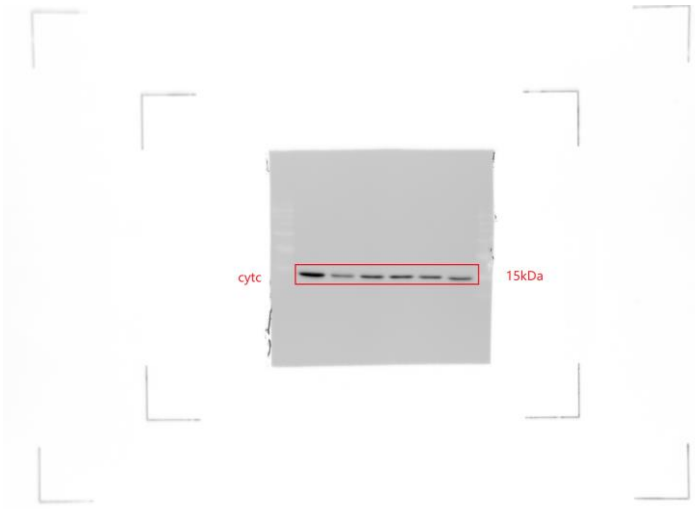

Apaf-1

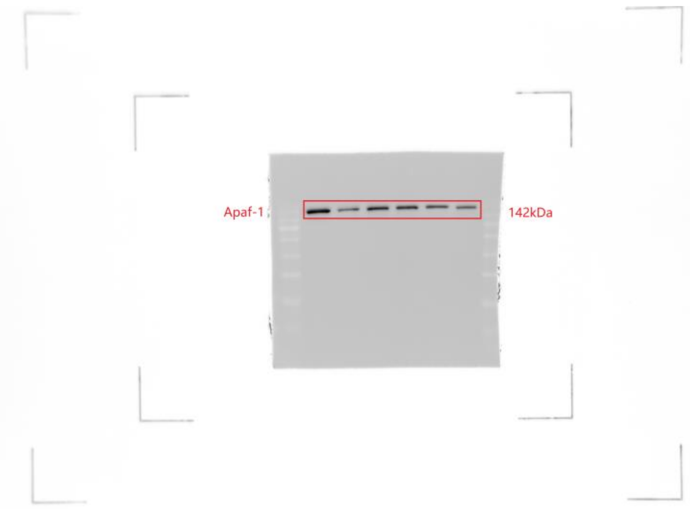

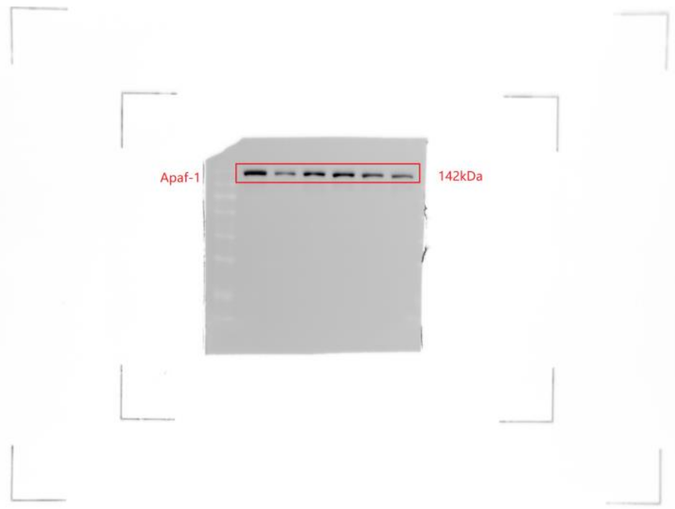

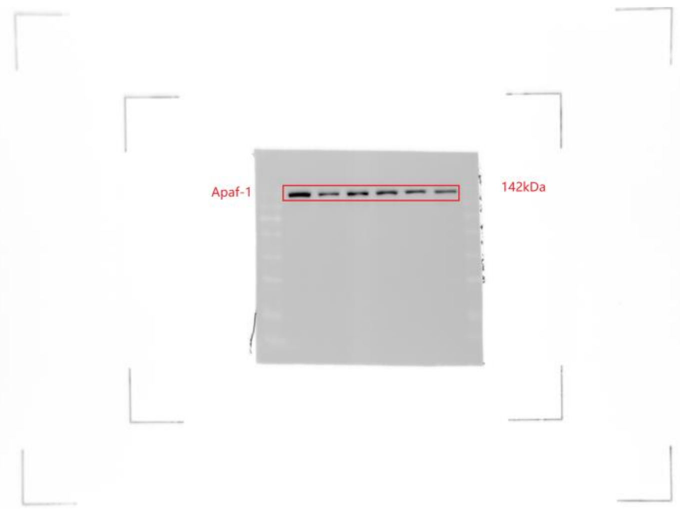

β-actin (Part 2)

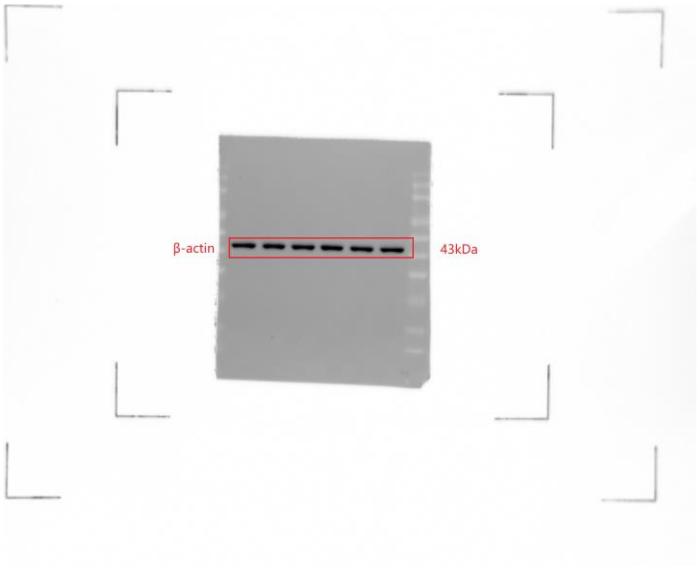

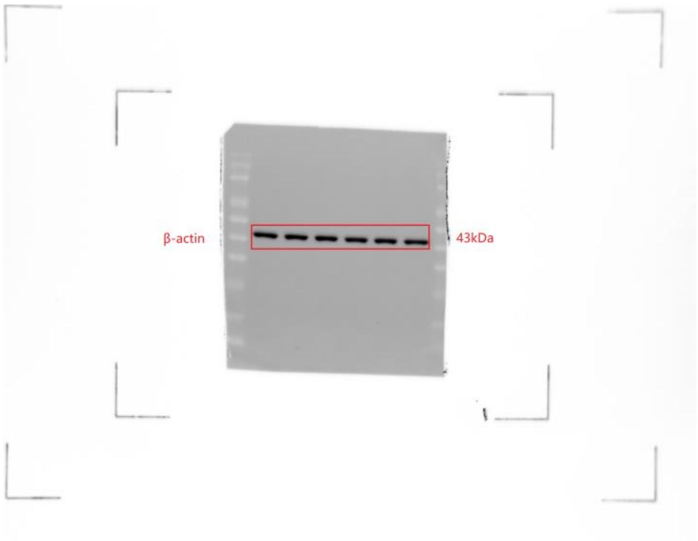

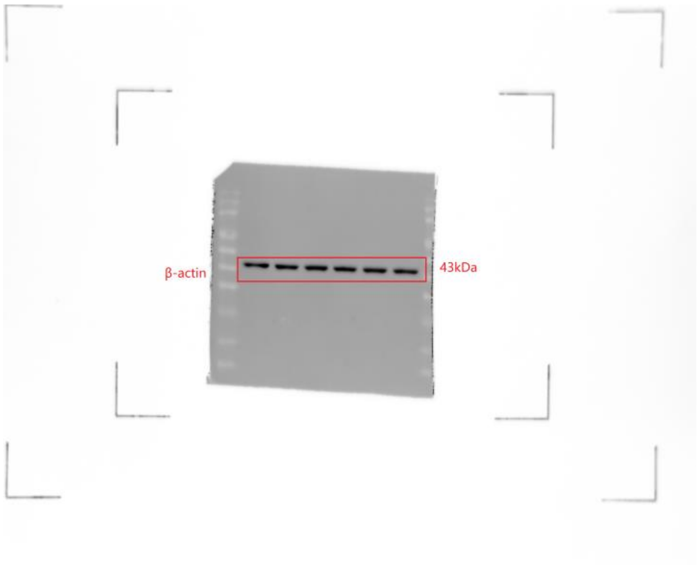

Caspase-3

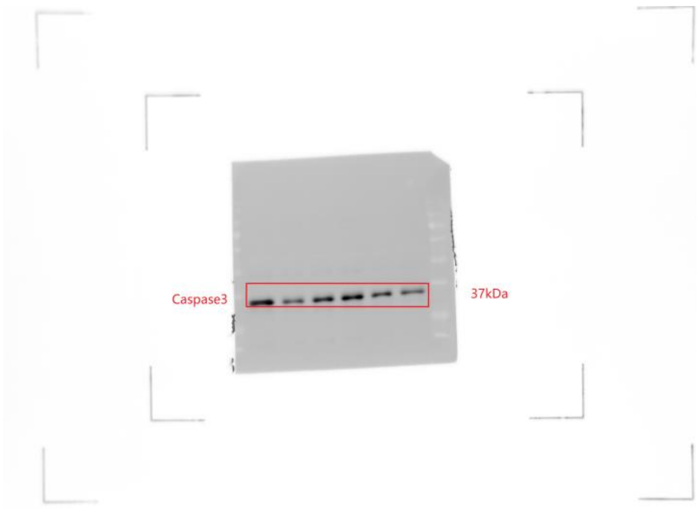

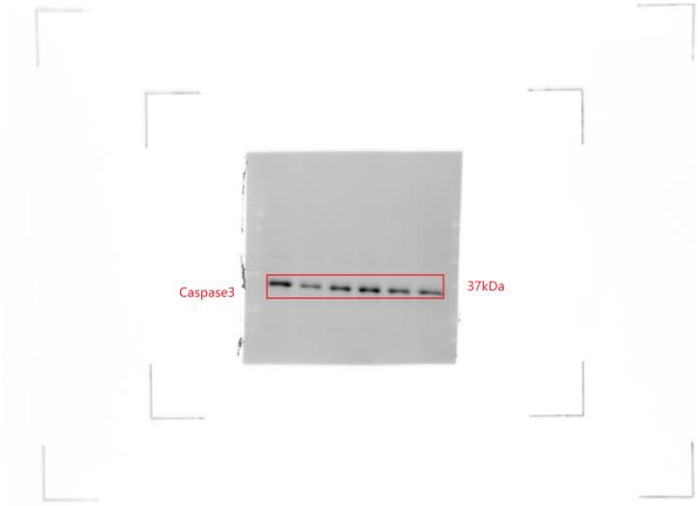

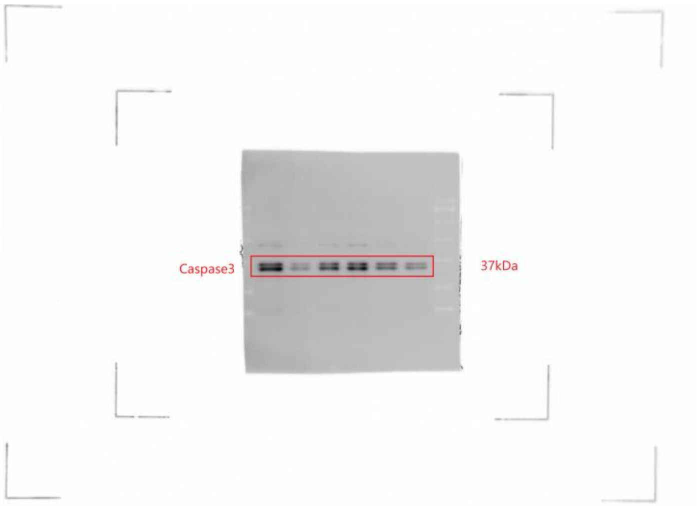

Cleaved Caspase-3

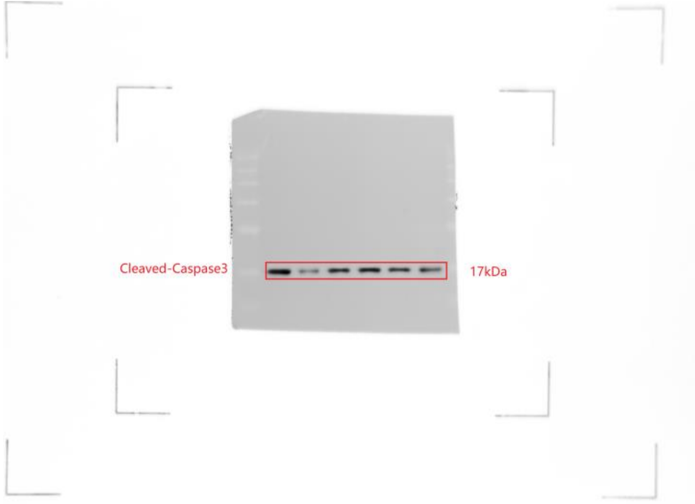

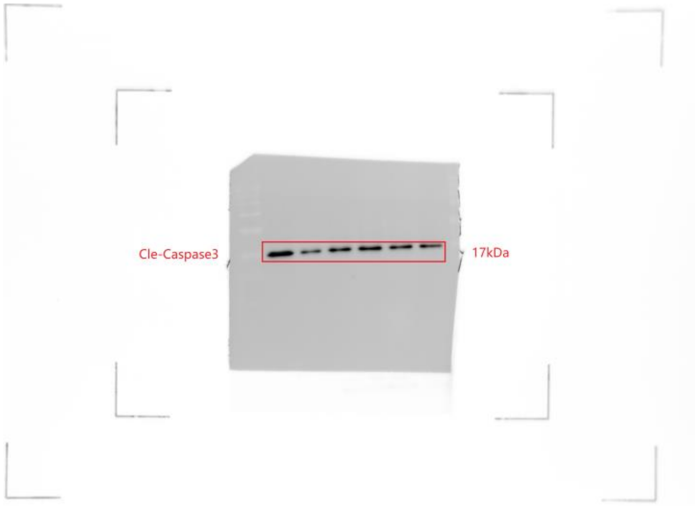

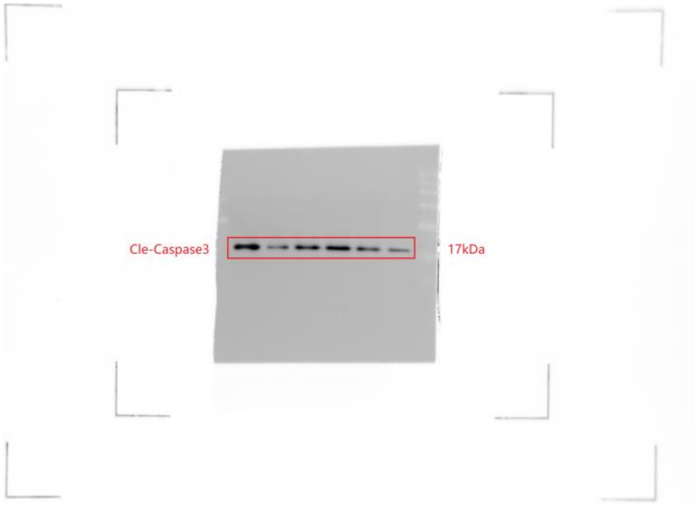

β-actin (Part 3)

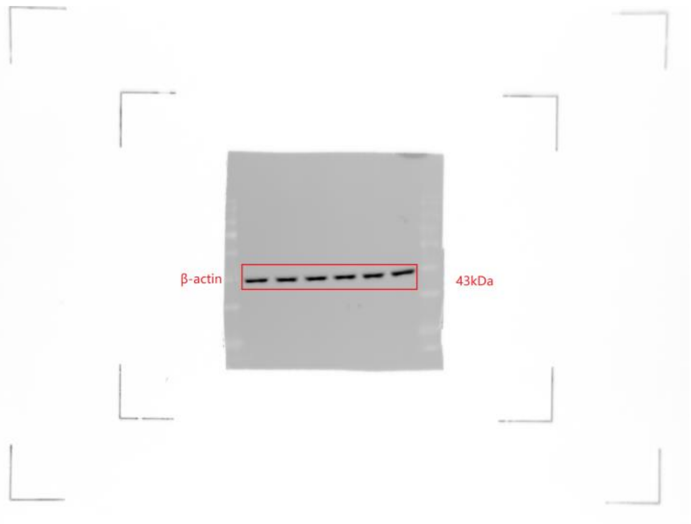

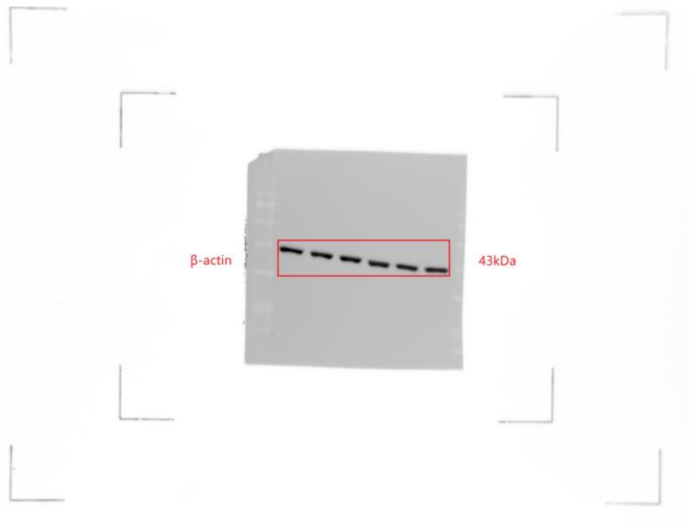

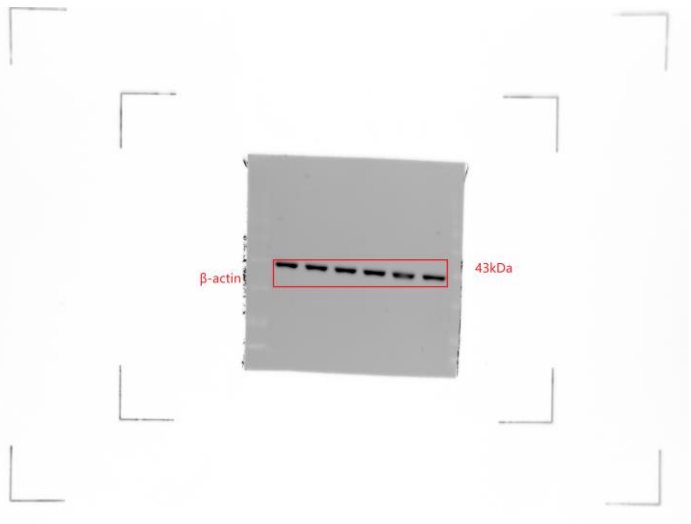

Supplement: Supplementary file 1 — Data S1. [file CNS-31-e70209-s002.pdf]
