# Supplementary material for: Investigation of Anti‐Apoptotic Effects and Mechanisms of Astragaloside IV in a Rat Model of Cerebral Ischemia–Reperfusion Injury
Source: CNS Neurosci Ther. 2025 Jan 7;31(1):e70209. doi: 10.1111/cns.70209 (PMC11705586; doi:10.1111/cns.70209)
Supplement: Supplementary file 2 — Data S2. [file CNS-31-e70209-s001.docx]

**Garcia JH scoring method**

1. **Spontaneous Activity**

The animal was observed for 5 min in its normal environment (cage). The rat’s activity was assessed by its ability to approach all four walls of the cage.

The scoring criteria are as follows: 3, rat moved around, explored the environment, and approached at least three walls of the cage; 2, slightly affected rat moved about in the cage but did not approach all sides and hesitated to move, although it eventually reached at least one upper rim of the cage; 1, severely affected rat did not rise up at all and barely moved in the cage; and 0, rat did not move at all.

1. **Symmetry in the Movement of Four Limbs**

The rat was held in the air by the tail to observe symmetry in the movement of the four limbs.

The scoring criteria are as follows: 3, all four limbs extended symmetrically; 2, limbs on left side extended less or more slowly than those on the right; 1, limbs on left side showed minimal movement; and 0, forelimb on left side did not move at all.

1. **Forepaw Outstretching**

The rat was brought up to the edge of the table and made to walk on forelimbs while being held by the tail. Symmetry in the outstretching of both forelimbs was observed while the rat reached the table and the hindlimbs were kept in the air.

The scoring criteria are as follows: 3, both forelimbs were outstretched, and the rat walked symmetrically on forepaws; 2, left side outstretched less than the right, and forepaw walking was impaired; 1, left forelimb moved minimally; and 0, left forelimb did not move.

1. **Climbing**

The rat was placed on the wall of a wire cage. Normally the rat uses all four limbs to climb up the wall. When the rat was removed from the wire cage by pulling it off by the tail, the strength of attachment was noted.

The scoring criteria are as follows: 3, rat climbed easily and gripped tightly to the wire; 2, left side was impaired while climbing or did not grip as hard as the right side; and 1, rat failed to climb or tended to circle instead of climbing.

1. **Body Proprioception**

The rat was touched with a blunt stick on each side of the body, and the reaction to the stimulus was observed.

The scoring criteria are as follows: 3, rat reacted by turning head and was equally startled by the stimulus on both sides; 2, rat reacted slowly to stimulus on left side; and 1, rat did not respond to the stimulus placed on the left side.

1. **Response to Vibrissae Touch**

A blunt stick was brushed against the vibrissae on each side; the stick was moved toward the whiskers from the rear of the animal to avoid entering the visual fields.

The scoring criteria are as follows: 3, rat reacted by turning head or was equally startled by the stimulus on both sides; 2, rat reacted slowly to stimulus on left side; and 1, rat did not respond to stimulus on the left side.

The score given to each rat at the completion of the evaluation is the summation of all six individual test scores. The minimum neurological score is 3 and the maximum is 18.

Table1. Key points of Garcia JH scoring criteria

| Test | Score | | | |
| --- | --- | --- | --- | --- |
|  | 0 | 1 | 2 | 3 |
| Spontaneous activity (in cage for 5 min) | No movement | Barely moves | Move and touch one side of cage (less than three sides) | Moves and approaches at least three sides of cage |
| Symmetry of movements (four limbs) | Affected side: no movement | Affected side: slight movement | Affected side: moves slowly | Both sides: move symmetrically |
| Symmetry of forelimbs (outstretching while held by tail) | Affected side: no movement, no outreaching | Affected side: slight movement to outreach | Affected side: moves and outreaches less than unaffected side | Symmetrical outreach |
| Climbing wall of wire cage | / | Fails to climb | Affected side: weak response | Normal climbing |
| Reaction to touch on either side of trunk | / | Affected side: no response | Affected side: weak response | Symmetrical response |
| Response to vibrissae touch | / | Affected side: no response | Affected side: weak response | Symmetrical response |
